# The Toxicity of Mercury and Its Chemical Compounds: Molecular Mechanisms and Environmental and Human Health Implications: A Comprehensive Review

**DOI:** 10.1021/acsomega.3c07047

**Published:** 2024-01-22

**Authors:** Yuan-Seng Wu, Ahmed I. Osman, Mohamed Hosny, Ahmed M. Elgarahy, Abdelazeem S. Eltaweil, David W. Rooney, Zhonghao Chen, Nur Syafiqah Rahim, Mahendran Sekar, Subash C. B. Gopinath, Nur Najihah Izzati Mat Rani, Kalaivani Batumalaie, Pow-Seng Yap

**Affiliations:** ∇ Sunway Microbiome Centre and Department of Medical Education, School of Medical and Life Sciences, Sunway University, 47500 Selangor Malaysia; # School of Chemistry and Chemical Engineering, Queen’s University Belfast, Belfast BT9 5AG, Northern Ireland, U.K.; 3 Green Technology Group, Environmental Sciences Department, Faculty of Science, Alexandria University, 21511 Alexandria, Egypt; 4 Egyptian Propylene and Polypropylene Company (EPPC), Port Said 42526, Egypt; 5 Environmental Chemistry Division, Environmental Science Department, Faculty of Science, Port Said University, Port Said 42526, Egypt; 6 Department of Chemistry, Faculty of Science, Alexandria University, 21321 Alexandria, Egypt; 7 Department of Civil Engineering, Xi’an Jiaotong-Liverpool University, Suzhou 215123, China; 8 Department of Biology, Faculty of Applied Sciences, Universiti Teknologi MARA, Perlis Branch, Arau Campus, Arau 02600, Perlis, Malaysia; 9 Department of Pharmaceutical Life Sciences, Faculty of Pharmacy, Universiti Malaya, Kuala Lumpur 50603, Malaysia; 10 School of Pharmacy, Monash University Malaysia, Subang Jaya, 47500 Selangor, Malaysia; 11 Faculty of Chemical Engineering Technology, Universiti Malaysia Perlis (UniMAP), Arau, 02600 Perlis, Malaysia; 12 Institute of Nano Electronic Engineering, Universiti Malaysia Perlis, Kangar, 01000 Perlis, Malaysia; 13 Faculty of Pharmacy and Health Sciences, Royal College of Medicine Perak, Universiti Kuala Lumpur, Ipoh 30450, Perak, Malaysia; 14 Pre-University Programmes, Sunway College Johor Bahru, Jalan Austin Heights Utama, Taman Mount Austin, 81100 Johor Bahru, Johor, Malaysia

## Abstract

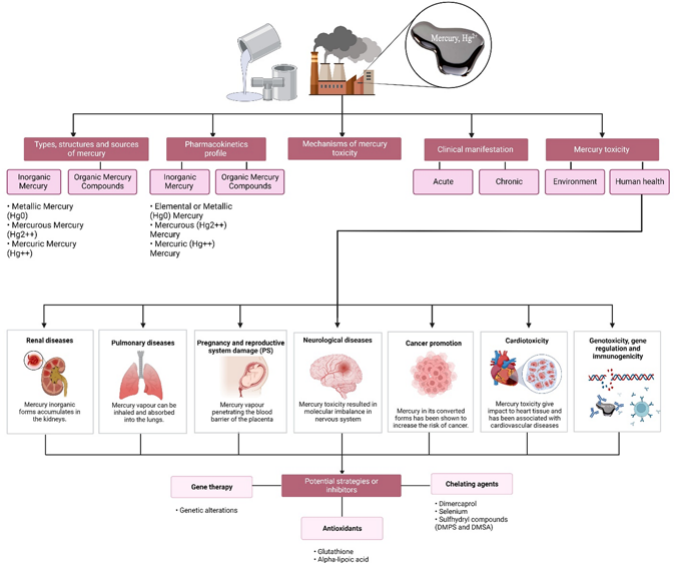

Mercury is a type of hazardous and toxic pollutant that can result in detrimental effects on the environment and human health. This review is aimed at discussing the state-of-the-art progress on the recent developments on the toxicity of mercury and its chemical compounds. More than 210 recent works of literature are covered in this review. It first delineates the types (covering elemental mercury, inorganic mercury compounds, organic mercury compounds), structures, and sources of mercury. It then discusses the pharmacokinetic profile of mercury, molecular mechanisms of mercury toxicity, and clinical manifestation of acute and chronic mercury toxicity to public health. It also elucidates the mercury toxicity to the environment and human health in detail, covering ecotoxicity, neurotoxicity diseases, neurological diseases, genotoxicity and gene regulation, immunogenicity, pregnancy and reproductive system damage, cancer promotion, cardiotoxicity, pulmonary diseases, and renal disease. In order to mitigate the adverse effects of mercury, strategies to overcome mercury toxicity are recommended. Finally, some future perspectives are provided in order to advance this field of research in the future.

## 1 Introduction

The chemical element mercury, abbreviated as “Hg”, is frequently found in the crustal rocks of the earth and in coal deposits.^1,2^ It is regarded as one of the most hazardous substances found on the surface of the earth.^3^ In general, there are four main ways that mercury can exist: as metallic elemental mercury (Hg^0^), as inorganic mercury (Hg^2+^), as methylmercury (MeHg), and as various organic molecules.^4^ Various sources can result in the release of mercury into the air that normally goes for chemical transformations in watercourses and soil, resulting in further toxicities on the environment and human health. These sources are mainly classified as natural and anthropogenic sources. Natural sources account for 5207 megagrams of mercury emissions annually, and it has to be mentioned that this amount is not purely released from natural sources, as it includes re-emissions of previously deposited mercury originating from anthropogenic and natural sources.^5^ Regarding anthropogenic sources, they account for 2320 megagrams of mercury emissions every year.^5^

**Figure 1:**
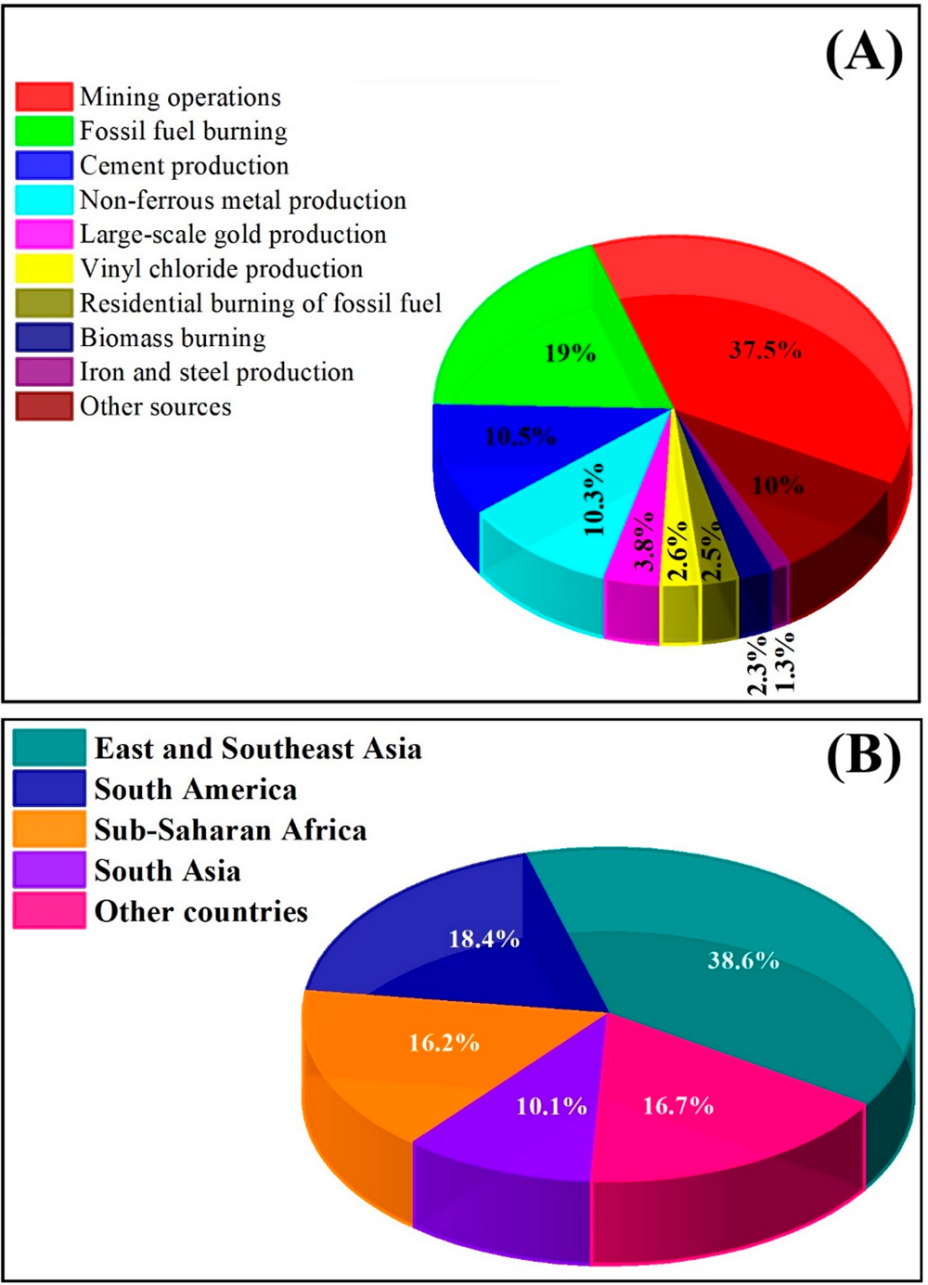
Sources of atmospheric mercury emissions (A) and contributing countries of atmospheric mercury emissions (B).

Also, it must be mentioned that most of these atmospheric emissions are deposited in soil and watercourses by dry and wet deposition and other chemical transformation processes.^1^ According to the global mercury assessment in 2018,^6^ mining operations are responsible for approximately 37.5% of air mercury emissions, followed by stationary industrial and power plants of fossil fuel burning (19%) along with stationary residential combustion of fossil fuels that account for 2.55%. Additionally, there are other sources of mercury, including cement production (10.5%), non-ferrous metal production (10.3%), large-scale gold production (3.8%), vinyl-chloride monomer (2.6%), biomass burning (2.3%), iron and steel production (1.3%), as well as other sources that are collectively contributing less than 10%, such as chlor-alkali production, oil refining, steel production, and waste incineration as shown in Figure 1A.

Based on the above-mentioned assessment report in 2018,^6^ East and Southeast Asia are reckoned the predominant contributors, as they are responsible for around 38.6% of the atmospheric mercury emissions, followed by South America (18.4%), Sub-Saharan Africa (16.2%), South Asia (10.1%), and the other world countries, including the European and Middle East countries, contributing less than 20%, as represented in Figure 1B.

The United States Environmental Protection Agency (EPA) has established rules that state that the maximum allowable level of mercury in water is 2 ppb,^7^ and unfortunately, the detected mercury concentrations in most of the watercourses exceed this limit. Also, it has to be mentioned that the safe dose of mercury in food is 0.1 μg/kg of body weight,^8^ but the actual concentration in most living organisms is higher. A maximum inhalation reference concentration of 0.3 μg/m^3^ for atmospheric Hg^0^ was previously set by the EPA, as well as reference doses of 0.3 μg/kg and 0.1 μg/kg per day for mercuric chloride and MeHg, respectively.^9^ Also, a concentration limit ranging from 0.2 to 1 mg/kg of MeHg in fish was previously set.^10^ Furthermore, a concentration limit of 0.07 to 0.3 mg/kg was determined for the total Hg concentration in the soil.^11^

Consequently, the EPA formulated standards for mercury and air pollution in 2011, which required coal-based industries to cut down on the release of harmful pollutants like mercury emissions.^12^ Additionally, one of the most fundamental international efforts to lessen mercury’s toxicity is the Minamata Convention on Mercury, which was signed in October 2013 and came into effect in 2017. This agreement aims to reduce the dangerous effects of mercury on both human health and the environment by addressing the entire life cycle of mercury, from mining and trade to use and disposal. To achieve this aim, different implementation procedures should be applied including limiting the sources of mercury discharge,^13^ regulating the supply of mercury to prevent its illegal trade,^14^ eliminating the use of mercury in artisanal and small-scale gold mining,^15^ promoting measures to reduce the demand for mercury, phasing out or restricting the manufacturing, import and export of products containing mercury,^16^ addressing the management of mercury-containing waste to prevent releases into the environment, and providing support to countries, especially developing countries, in building capacity for the sound management of mercury. The number of signatories to this convention was 84 in 2017 and reached 128 in March 2019, indicating the increasing global efforts to reduce mercury emissions into the environment and their negative impacts.

**Figure 2:**
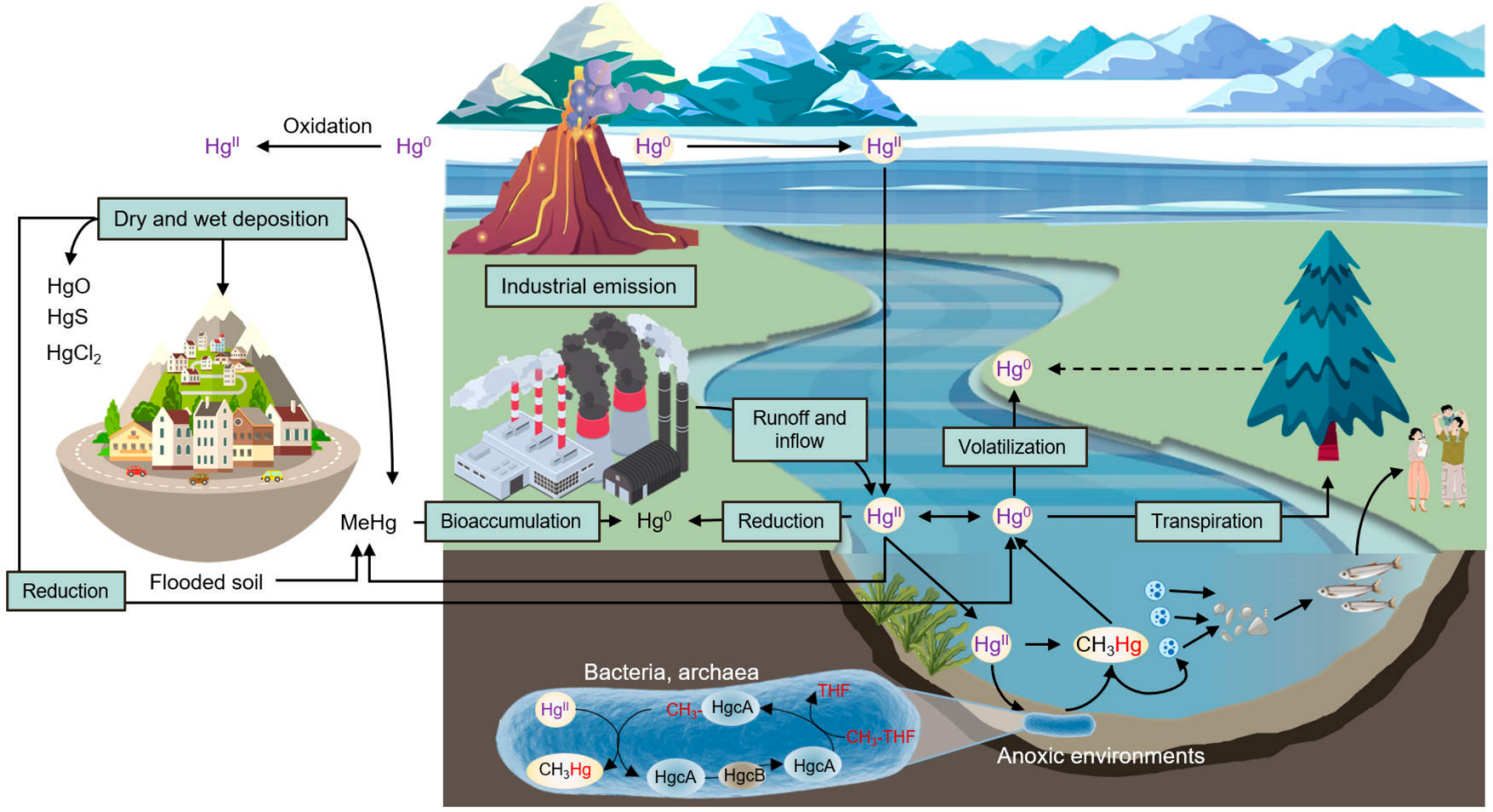
Biogeochemical cycle of mercury in the atmosphere, soil, and aquatic environment.

The biogeochemical cycle of mercury involves the movement and transformation of mercury through various environmental compartments, including the atmosphere, soil, and aquatic systems, as shown in Figure 2. This cycle is complex and is influenced by both natural processes and human activities. Here is a detailed explanation of each stage in the biogeochemical cycle of mercury:

In the atmospheric phase, mercury exists in the atmosphere primarily as elemental mercury (Hg(0)) vapor, which is released through natural sources such as volcanic emissions, forest fires, and weathering of rocks. Anthropogenic activities, such as coal combustion and industrial processes, also contribute to atmospheric mercury levels. Once in the atmosphere, mercury can travel long distances and undergo global-scale distribution through atmospheric transport.^17^ While, in wet and dry deposition, mercury in the atmosphere can undergo wet and dry deposition processes. Wet deposition occurs when mercury is scavenged from the atmosphere by precipitation (rain or snow) and deposited on land or water surfaces. Dry deposition involves the direct deposition of gaseous or particulate mercury onto surfaces without precipitation. Both wet and dry deposition contribute to the input of mercury into soil and water systems.^18^

Moreover, in soils, mercury can undergo several processes. Part of the deposited mercury can be converted to inorganic mercury (Hg(II)) through oxidation reactions mediated by microbial activity, sunlight, and chemical processes. Hg(II) can bind to soil particles or be transformed into methylmercury (MeHg) through microbial methylation. MeHg is a highly toxic form of mercury that can bioaccumulate up the food chain.^19^ Mercury can enter aquatic systems through runoff from soil, atmospheric deposition, and direct discharge from point sources. In water bodies, mercury can undergo transformation processes. Inorganic mercury can be converted to MeHg by certain anaerobic bacteria present in sediments and water. MeHg can accumulate in aquatic organisms, particularly in fish and other aquatic organisms at higher trophic levels.^20^

Furthermore, mercury bioaccumulates in aquatic organisms as they take up MeHg from the water or ingest prey containing MeHg. This process leads to higher concentrations of mercury in organisms at higher trophic levels due to biomagnification. As a result, predatory fish and marine mammals can accumulate significant levels of mercury.^21^

Humans can be exposed to mercury through the consumption of contaminated fish and seafood. When humans consume fish containing MeHg, it can be absorbed into their bodies and distributed to various tissues, including the brain, where it can cause adverse health effects.^22^ Mercury can also undergo volatilization from water and soil surfaces back into the atmosphere. This process completes the biogeochemical cycle of mercury, allowing it to be transported and redistributed to different environmental compartments.^23^

Overall, the biogeochemical cycle of mercury involves complex interactions among the atmosphere, soil, and aquatic systems, with transformations between different chemical forms and potential for bioaccumulation and biomagnification. Human activities, particularly those involving the release of mercury into the environment, can significantly impact this cycle and have implications for both environmental and human health.

## 2 Types, Structure, and Sources of Mercury and Pharmacokinetic Profile of Mercury

Mercury is commonly known as quicksilver, and it is a unique element with distinctive properties. It possesses an atomic mass of 200.59 g/mol and an atomic number of 80, making it the only metal that remains in the liquid state at room temperature. Its specific gravity is about 13.5 times that of water. Notably, mercury has a relatively low melting point of −38.8 °C and a boiling point of 356.7 °C.^24^

Mercury’s chemical behavior is quite intriguing. Depending on factors such as its chemical form, dose, exposure duration, and pathway, it can have varying degrees of harmful effects on human tissues and organs. It is categorized as a “soft sphere” in Ralph Pearson’s HSAB (Hard and Soft Acids and Bases) classification due to its outer shell’s highly polarizable electrons. This property leads to a strong attraction to ligands containing soft donor atoms, including sulfur (S), selenium (Se), phosphorus (P), and halide ions like iodide (I^–^), bromide (Br^–^), and chloride (Cl^–^). Compounds with thiol groups, referred to as mercaptans, have a particular affinity for Hg^2+^.^25^

Mercury interacts with a range of substances, including amino acids, peptides, proteins, dissolved organic matter, and thiol-containing pharmaceutical agents, which results in the formation of different organic mercury compounds. Additionally, the photochemical reduction of ionic Hg to elemental Hg (Hg^0^) followed by its reoxidation to ionic Hg^26^ can impact its volatilization and bioavailability to living organisms.

The solubilities of various mercury compounds varies. Hg^0^ is insoluble in water, while compounds such as Hg(I) chloride and mercury sulfide (HgS) exhibit limited solubility. In contrast, Hg(II) chloride readily dissolves. For instance, HgS and mercury hydroxide (Hg_2_(OH)_2_) have low solubility product (*K*_sp_) constants of 10^–23.7^ and 10^–52^, respectively,^27^ affecting their solubility in different environmental conditions.

In biological pathways, mercury undergoes both oxidation and reduction reactions, resulting in changes to its speciation and influencing its biological uptake.^28^ The interconversion of different mercury species plays a significant role in regulating its behavior.

Mercury can be classified into (i) inorganic mercury and (ii) organic mercury. These distinct forms of mercury exhibit varying properties and behaviors in environmental and biological contexts.

### 2.1 Inorganic Mercury

Inorganic mercury has a low bioavailability via the oral route, with absorption rates ranging from 7% to 15% based on the amount of inorganic mercury consumed. It has been reported that the highest quantities of inorganic mercury are detected in the kidney.^29^ Hg^0^ (metallic), Hg_2_^2+^ (mercurous), and Hg^2+^ (mercuric) mercury are the three states of inorganic mercury. Mercury’s pharmacokinetics and biotransformation are determined by its chemical and physical state.^30^

The toxic potential of mercury salts is influenced by their solubility. In general, mercurous compounds exhibit lower toxicity compared to mercuric compounds due to their reduced solubility in water.^31^ Mercury salts exhibit greater corrosiveness compared to elemental mercury, leading to increased gastrointestinal permeability and absorption.^31^

Chronic exposure to Hg^0^ results in vomiting, abdominal pain, renal tubular necrosis, emotional changes, and cognitive deficits, whereas severe lung and neurological damage is caused by acute exposure to Hg^0^, as further detailed in Table 1. The toxicological effects of mercurous compounds include abdominal pain, nausea, vomiting, toxicity of blood, damage to the central nervous system, memory disturbances, fatigue, muscle weakness, and kidney damage.^32^ Additionally, human exposure to mercuric compounds causes numerous issues such as memory and concentration troubles, irritation of skin and eyes, toxicity of the reproductive system of both males and females, malfunction of the immune system, loss of appetite and weight, and changes in mood and personality.^33^ Further details about the toxic effects of each of these mercury compounds are provided in Table 1.

**Table 1:** Physicochemical Properties of Most Common Hg Compounds^24,31−33,53−56^

Name	Chemical Formula	Toxicological Effects	Density (g/cm^3^)	Solubility in Water (g/L)	Vapor Pressure (Pa)	Temperature of Decomposition or Sublimation
Elemental mercury	Hg^0^	Vomiting, abdominal pain, and renal tubular necrosis.	13.53 (25.0 °C)	5.6 × 10^–5^ - 6.1 × 10^–5^ (25.0 °C)	0.27 (25.0 °C)	38.8 °C (m.p), 356.7 °C (b.p.)
		Tremors, emotional changes, and cognitive deficits are caused by chronic exposure, whereas severe lung and neurological damage are caused by acute exposure.				
Mercurous oxide	Hg_2_O	Abdominal pain, nausea, vomiting, and diarrhea upon ingestion.	9.80 (25.0 °C)	Insoluble	-	100 °C (decomposes to elemental mercury)
		Long-term or chronic exposure can affect the central nervous system.				
Mercuric oxide	HgO	Memory and concentration troubles, gum problems, increased salivation, loss of appetite and weight, and changes in mood and personality.	11.14 (25.0 °C)	0.043–0.053 (25.0 °C)	9.20 × 10^–12^ (25.0 °C)	500 °C (m.p.)
Mercury sulfide (red)	HgS	Negatively affecting the immune system after being absorbed by the gastrointestinal tract, and accumulated in the spleen and thymus.	8.10 (25.0. °C)	2.0 × 10^–24^ (25.0 °C)	-	344 °C (transform to black HgS), 584 °C (sublimation)
Mercurous chloride	Hg_2_Cl_2_	Sensory and memory disturbances, fatigue, muscle weakness and kidney damage.	5.43 (25.0 °C)	28.6–73.3 (25.0 °C)	9.00 × 10^–3^ (20.0 °C)	383 °C (sublimation)
Mercuric chloride	HgCl_2_	Negatively affecting peripheral vision, skin allergy, and also hallucinating and psychosis in extreme cases.	5.43 (25.0 °C)	28.6–73.3 (25.0 °C)	9.00 × 10^–3^ (20.0 °C)	277 °C (m.p.), 304 °C (b.p.)
Mercurous sulfate	Hg_2_SO_4_	Toxicity of blood, kidney, lungs, and central nervous system.	7.56 (25.0 °C)	0.51 (25.0 °C)	-	450 °C (decomposition)
Mercuric sulfate	HgSO_4_	Tightness in the chest, difficulties breathing, coughing and pain.	6.47 (25.0 °C)	Decomposes to mercury oxide sulfate and sulfuric acid	-	335–500 °C (decomposition)
		Ulceration of conjunctiva and cornea.				
Mercuric Fluoride	HgF_2_	Highly toxic upon ingestion, inhalation, and skin absorption, and also causes toxicity of the reproductive system.	8.95 (25.0 °C)	Reacts^57^	-	645 °C (decomposition)
Mercuric bromide	HgBr_2_	Highly toxic and causes kidney damage.	6.03 (25.0 °C)	220 (250 °C)		237 °C (m.p.), 322 °C (b.p.)
Mercuric iodide	HgI_2_	Skin irritation and eye damage.	7.15 (25.0 °C)	0.0002 (250. °C)	-	259 °C (m.p.), 350 °C (b.p.)
Mercuric cyanide	Hg(CN)_2_	Oxidative stress, lipid peroxidation, mitochondrial dysfunction.	4.00 (25.0 °C)	93.0 (14.0 °C)	-	320 °C (decomposition)
Mercuric selenide	HgSe	Neurotoxicity and reproductive toxicity of both males and females.	8.27 (25.0 °C)	Insoluble	-	997 °C (m.p.)
Mercuric acetate	Hg(CH_3_COO)_2_	Irritation and damage to eyes.	3.28 (25.0 °C)	Soluble	0.24 (25.0 °C)	179 °C (decomposition)
		Irritation of nose, throat, and lungs.				
		Skin allergy.				
Mercuric nitrate	Hg(NO_3_)_2_	Irritation of skin and eyes.	4.30 (25.0 °C)	Soluble	0.24 (25.0 °C)	79 °C (m.p.)
		Damage of gastrointestinal tract.				
		Kidney failure.				
Methyl mercury	CH_3_Hg^+^	Toxicity of the central and peripheral nervous systems.	1.08 (25.0 °C)	Very low (less than 0.01 g/L)	10^–11^ (25.0 °C)	300–400 °C (decomposes to mercury vapor)
		Memory dysfunction and attention deficits.				
		Brain damage.				
		Hearing impairment, blindness, and death.				
Dimethyl mercury	(CH_3_)_2_Hg	Severe neurological damage and death.	3.19 (20.0 °C)	Slightly soluble	8.31 × 10^3^ (25.0 °C)	–43 °C (m.p.), 5 °C (flash point), 93–94 °C (b.p.)
Ethyl mercury	C_2_H_5_Hg	kidney damage, and digestive tract problems, including diarrhea, nausea, and ulcers.	1.08 (25.0 °C)	Very low	-	Above 200 °C
Methyl mercuric chloride	CH_3_HgCl	Skin burns, nausea, abdominal pain, vomiting, and diarrhea.	4.06 (200. °C)	<0.10 (210. °C)	1.10–1.76 (25.0 °C)	170–173 °C (m.p.)
Methyl mercuric hydroxide	CH_3_HgOH	Neurotoxicity.	-	1.0 to 10.0 (21.0 °C)	0.90	N/A

#### 2.1.1 Metallic Mercury (Hg^0^)

There are several applications for metallic or elemental mercury (Hg^0^), which has no electrical charge. The Hg^0^ outgassed from amalgams enters the body by ingestion, absorption through the skin, or breathing in the event of direct contact. Around 80% of the metallic mercury vapor generated by amalgams is inhaled, compared to 7% to 10% that is consumed and just 1% that is absorbed by skin contact. Then afterward, it forms bonds with sulfur-containing amino acids. This Hg^0^ vapor reaches the brain in serum (dissolved) or as an adherent to the membrane of red cells, where it dwells in the fetal brain. In addition, Hg^0^ lodges in many organs, including the breast, muscles, thyroid, lungs, liver myocardium, kidneys, prostate, skin, pancreas, sweat glands, enterocytes, testes, and salivary glands, which causes many problems and may lead to their dysfunction.^34^ Although Hg^0^ oxidizes rapidly in the bloodstream, the absorption rate of metallic mercury by the central nervous system is faster than its oxidation.

Mercury also strongly afflicts T cell surface binding sites and sulfhydryl groups, influencing T cell function. The majority of metallic mercury is passed off as mercuric mercury. The excretory half-life of metallic and mercuric mercury can vary from a few days to several months depending on the organ of deposition and redox state, with certain pools (such as the Central Nervous System (CNS)) having a half-life of up to many years. Mercury in the hair has no relationship to the quantity of metallic mercury in the brain. Therefore, the accurate measurement of the body load is challenging.^34,35^

Mercuric sulfide (HgS) is employed to extract the metallic form of Hg.^36^ However, the Hg^0^ is available in the liquid form (i.e., silver-colored liquid) under ambient conditions; it may form Hg vapors under the same environmental conditions (i.e., room temperature), attributing to its high vapor pressure.^37^ Referring to its chemical properties, Hg is particularly applicable to be employed in various industries such as the extraction of gold and silver from ores,^38^ dental amalgam,^39^ and others. In nature, mercury mostly forms compounds with the functional groups OH^–^, Cl^–^, and S^2–^ as well as with organic ligands.^28,40^

#### 2.1.2 Mercurous Mercury (Hg_2_^2+^)

Chemically, Hg is regarded as a cation with different oxidation states of monovalent Hg^1+^ (i.e., mercurous cation, stable form Hg_2_^2+^) or divalent Hg^2+^ (i.e., mercuric cation, Hg^2+^).^41^ Most Hg_2_^2+^ salts are poorly soluble in water, including Hg_2_Cl_2_ and Hg_2_SO_2_. Under normal conditions, the Hg_2_^2+^ is unstable and tends to transform into Hg^0^ and Hg^2+^ via the dismutation process.^27,42^ Moreover, it may interact with common metal ions (i.e., chloride, halides, and sulfide) and produce compounds with very low solubility characters.^43^

The mercurous mercury salt Hg_2_Cl_2_ (calomel) is poorly soluble in water and poorly absorbed by the gut, but some of it may oxidize to forms that are more readily absorbed. Mercuric mercury does not often remain in the body unless it is in a condition between metallic and mercuric mercury. It is infrequently linked to pink illness or acrodynia, indicating that some absorption occurs.^34^

#### 2.1.3 Mercuric Mercury (Hg^2+^)

Hg^2+^ is the most stable form of mercury in aqueous solutions.^44^ The most common inorganic species of Hg^2+^ comprise mercuric sulfide (HgS), mercuric oxide (HgO), mercuric sulfate (HgSO_4_), and mercuric chloride (HgCl_2_). Historically, HgCl_2_ was used to preserve and develop the photographic film and as a gradient in some skin-lightening creams. Hg^2+^ is hardly absorbed by the body where ≤2% of ingested HgCl_2_ could be absorbed;^45^ however, its permeability may be increased as a result of its corrosive effect on the intestine with extended exposure.^46^ Hg^2+^ adheres to S-containing amino acids in the circulation with the same affinity as Hg^0^. Hg^2+^ builds up in the amniotic fluid, fetal tissues, and placenta even though it was unable to effectively penetrate the blood-brain barrier.^47^

Hg^2+^ is dispersed by one or more amino acid transporters, particularly cysteine transporters, according to the evidence, which may help to explain why it concentrates in the brain.^48^ In the kidneys, the proximal convoluted renal tubule, which is bound to metallothionein, stores a large amount of mercuric compounds in the body. Smaller amounts of mercuric comounds are found in epithelial tissues, the choroidal plexus, and the testes. Periportal deposition of mercuric compounds is also frequent in the liver. Small amounts of mercury can also be found in saliva, tears, breast milk, perspiration, and feces. Mercuric mercury is primarily expelled through urine and feces. Previous investigations have shown an effective half-life of 42 days for 80% of an oral tracer dosage, demonstrating that its half-life appears to be multiphasic. The excretion rate of the remaining 20% does not seem to be quantifiable.

### 2.2 Organic Mercury Compounds

Organic mercury has been reported to effectively get absorbed through the lungs when consumed compared to only trace quantities absorbed into the skin. Methyl mercury (MeHg), a major source of mercury exposure, is primarily found in contaminated food and is being exposed to some susceptible groups around the world in the specific form of these metal–organic mercury complexes. MeHg and other organic forms of mercury are particularly hazardous to humans because of their long-term toxicity and ability to cross any cellular barrier.^49^ Following the Minamata incident in Japan in 1956, which led to the identification of Minamata disease resulting from the consumption of fish and shellfish contaminated with methylmercury, numerous scientists initiated research on the process of demethylating methylmercury in the human body.

The neurological system, bone marrow, kidneys, brain, placenta, and fetus, in especially the fetal brain, all have significant quantities of MeHg.^50^ Over time, MeHg builds up and is demethylated to inorganic mercury. In 1986, Tsubaki et al.^51^ conducted a study in which they measured the levels of total mercury and MeHg in the brains of approximately 30 human autopsy cases in Japan. These individuals passed away between 20 days and 18 years after experiencing the onset of symptoms related to MeHg poisoning. In cases classified as “acute” (meaning autopsy conducted within 100 days of the onset of symptoms), the total mercury content in the brain ranged from 8.8 to 21.4 mg Hg/kg (measured on a wet weight basis), while the levels of MeHg were between 1.85 and 8.42 mg Hg/kg. For the “chronic” cases (autopsy performed 100 days to 18 years after symptom onset), the corresponding brain concentrations were lower, with total mercury ranging from 0.35 to 5.29 mg Hg/kg and MeHg ranging from 0.31 to 1.02 mg Hg/kg. On average, only 28% of the total mercury was found to be in the form of MeHg in the acute cases, and this percentage dropped to 17% in the chronic cases. Such results indicated that the remaining portion is inorganic mercury, and the difference indicated that there may be an ongoing process of demethylation of MeHg in the brain.

In humans, MeHg has a half-life of around 70 days, with most of it being excreted in the feces (about 90%) and some being transported through the enterohepatic system. The amount released in breast milk, which varies depending on the level of exposure, is about 20% MeHg. MeHg has a short half-life in the blood, making it inappropriate for calculating the total body burden similarly to Hg^0^. Additionally, dimethyl mercury is easily absorbed via the skin, and death could be caused just by having minimal skin contact with it.^48,52^

Ethyl mercury behaves similarly to MeHg at the cellular level but has a one-third longer excretory half-life. MeHg vapor has an identical (80%) absorption efficiency as metallic mercury vapor.^52^ MeHg attaches to sulfhydryl groups, especially those present in cysteine, as it enters the bloodstream. After that, it is dispersed throughout the body, and 4 days after exposure, blood and body homeostasis are restored. The sulfhydryl group in cysteine is likely connected to one or more transporters, primarily the cysteine transporter, which appears to be in charge of distribution to various bodily areas.

Both methylmercury (CH_3_Hg^+^) and dimethylmercury ((CH_3_)_2_Hg) are conceived as the most widely Hg-based compounds in the environment, majorly resulting from the methylation of Hg^2+^ by microorganisms within soil and water and hence bioaccumulate through the food chain. Chiefly, the reaction between different organic ligands and Hg produces R-Hg^+^ and R-Hg-R compounds. Another type of organic mercury, Hg-amino acid molecular interactions, is related to the amino acid Cys. Among the different Hg complexes, Hg(Cys)_2_ is the prevalent one with the ability to surpass the cell membrane. Therefore, the complex [Hg(Cys)_2_] is proposed to imitate Cys and employ the active cell transport sites normally associated with Cys transport across the membrane.^43^Table 1 demonstrates various examples of Hg compounds with their physicochemical properties.

To sum up, Hg can exist in various chemical forms, and its interactions with different ligands depend on its “soft sphere” nature, especially with soft donor atoms like S, Se, P, and halide ions. In aquatic environments, it tends to bind with DOM, particularly compounds containing thiol groups and other sulfur-containing compounds. The volatilization loss and bioavailability of mercury are affected by its photochemical reduction to elemental mercury and reoxidation back to ionic mercury. Mercury’s speciation significantly influences its biological behavior, and it can be categorized into inorganic mercury (Hg^0^, Hg^2+^), and organic mercury compounds (e.g., MeHg). In general, inorganic mercury has low bioavailability via oral routes, while organic mercury is more toxic and can accumulate in the neurological system and other tissues.

## 3 Clinical Manifestation of Acute and Chronic Mercury Toxicity to Public Health

Different mercury compounds can cause various clinical symptoms. Mercury poisoning frequently results in a false diagnosis because of its slow onset and ambiguous clinical symptoms. The quantity, length, and mode of exposure all affect how clinically a person who has been exposed to mercury will present. The most frequent cause of acute poisoning is inhalation of elemental mercury or ingestion of inorganic mercury. Chronic poisoning is more likely to result from exposure to organic mercury. Regardless of the type of mercury present, the two main organs affected by poisoning are the kidneys and the central nervous system. Nevertheless, the kidneys are home to practically all mercury compounds.^58^

### 3.1 Clinical Signs of Acute Exposure

Acute toxicity to elemental mercury by inhalation can cause respiratory symptoms. Inhaling large quantities of mercury vapor causes interstitial pneumonitis and acute corrosive bronchitis. Acute exposure can cause cough with fever, shortness of breath, headache and muscular pains.^59^ Early clinical signs, including shortness of breath, fever, chills, taste of metal, and pleuritic chest pain, may be mistaken for metal fume fever. Other potential clinical manifestations include stomatitis, lethargy, confusion, and vomiting. Although the healing process is possible, inhaled exposure can also cause pulmonary problems such as pneumothorax, interstitial emphysema, pneumatocele, interstitial fibrosis, and pneumomediastinum. In addition, exposure to extremely high levels of elemental mercury might result in lethal acute respiratory distress syndrome.^58,60^

The most common route of acute exposure to inorganic mercury or mercuric salt is through the mouth. These chemicals’ corrosive characteristics cause most of the acute clinical manifestations of poisoning. Acute symptoms may include ashen-gray mucous membranes due to mercuric salt precipitation, vomiting, hematochezia (bloody stool), hypovolemic shock, and severe abdominal pain. Systemic effects typically appear several hours after administration and might linger for many days. These negative consequences include dental sensitivity, mouth soreness, foul smell, mucosal inflammation, gingival irritation, and renal tubular necrosis, which can cause oliguria or anuria.^58,60^

### 3.2 Clinical Signs of Chronic Exposure

Chronic toxicity is typically caused by extended exposure of workers to elemental mercury that is transformed into the inorganic form. The CNS is the most affected by chronic mercury vapor exposure. The consequences may not be obvious initially, and early signs are nonspecific, known as an asthenic vegetative syndrome or micro-mercurialism.^61^ Chronic mercury poisoning is frequently brought on by the use of diuretics or cathartics that contain mercury over an extended period of time. Both chronic and high doses cause a range of psychiatric, kidney, neurological, and dermatological symptoms.^58,60,62^ Anorexia, weight loss, weariness, and physical weakness may occur in the exposed individual, and this clinical manifestation might suggest various disorders. The CNS is quickly penetrated by elemental mercury vapor and short-chain alkylmercury compounds, which bind to and inhibit synaptic and neuromuscular transmission-related proteins and enzymes. The blocking of these signals has the usual degenerative repercussions. As a result, the individual may have mild tremors in their hands and fingers, which may eventually spread to their entire leg.^58,60,62^

The classic triad of symptoms associated with chronic mercury toxicity is gingivitis, tremors, and erethism (a constellation of neuropsychiatric abnormalities that also includes memory loss, insomnia, sadness, shyness, emotional instability, anorexia, flushing, vasomotor disruption, and uncontrolled sweating). Peripheral neuropathy, headache, salivation, visual disruption, sleeplessness, and ataxia are possible clinical manifestations caused by mercury exposure.^58,60,62^ The clinical manifestations of organic mercury compound poisoning are similar to those of elemental mercury poisoning: unsteady walking, ataxia, illegible handwriting, and tremors. A loss of facial muscle tone can also cause slurred speech. A tiny fraction of people exposed to inorganic mercury have a widespread condition called acrodynia. Its symptoms include erythema of the soles and palms, irritability, edema of the hands and feet, hair loss, a desquamating rash, tachycardia, pruritus, diaphoresis, anorexia, hypertension, photophobia, sleeplessness, constipation or diarrhea, and decreased muscular tone. It is also known as Pink Disease. It was more frequent when diapers were washed in mercury-containing detergents or fungicides or when mercury-containing teething powders were used. The most frequent source of organic mercury poisoning is eating contaminated food, especially fish.^58,60,62^

Long-chain and aryl forms of organic mercury are equally as hazardous to humans as inorganic mercury. The motor and sensory centers, cerebral cortex, cerebellum, and auditory center are all targets for organic mercury. After exposure, symptoms frequently take days or weeks to manifest. Before symptoms manifest, the enzymes to which organic mercury binds must be degraded. Dysarthria, visual disturbances, ataxia, mental deterioration, paresthesias, hearing loss, muscular tremors, movement disorders, and paralysis and death are common toxicity symptoms in extreme cases. Mercury is hazardous to the fetus in any form, but methylmercury most easily penetrates through the placenta. Even in asymptomatic patients, maternal exposure might cause spontaneous miscarriage or retardation.^58,60,62^

In conclusion, ingesting inorganic mercury or mercuric salts can result in corrosive effects, leading to symptoms such as vomiting, abdominal pain, and renal tubular necrosis. Acute exposure to Hg^0^ through inhalation can cause respiratory symptoms and potentially lethal acute respiratory distress syndrome. While chronic exposure to the vapors of Hg^0^ affects the central nervous system, causing nonspecific early signs, like an asthenic vegetative syndrome. Chronic mercury poisoning from extended exposure can lead to psychiatric, kidney, neurological, and dermatological symptoms. Symptoms of chronic exposure include tremors, gingivitis, and erethism, while organic mercury compounds from contaminated food may cause dysarthria, visual disturbances, and paralysis. Mercury is also hazardous to the fetus, with MeHg easily crossing the placenta and causing potential miscarriage or retardation.

## 4 Mercury Toxicity on Human Health: Effects and Molecular Mechanisms (Table 2)

The largest health danger from Hg comes from human exposure to MeHg species through food. The main route of MeHg exposure is by ingestion of aquatic animals, mostly fish, and MeHg is distributed throughout human tissues via bloodstream absorption.^63,64^ The brain (central nervous system), kidneys, and liver are the primary organs in humans where MeHg builds up. Additional negative effects of high-dose Hg exposure include hearing loss, visual issues, speech impairments, neuronal cell death, and fatalities from life-threatening illnesses.^65,66^

Additionally, it is transferred to the placenta, which has a detrimental effect on how the child’s brain develops. As a result of MeHg exposure, it may be claimed that prenatal life is more susceptible compared to adult life. Hg toxicity depends on the amount and rate of exposure to various forms of Hg, with the brain being the primary target organ for breathed Hg vapor. Hg exposure in humans is determined by measuring Hg levels in hair, blood, and urine.^67,68^

### 4.1 Neurological Diseases

Depending on the individual molecule and exposure route, mercury has a variety of hazardous consequences.^69^ There are both inorganic and organic Hg compounds of this environmental toxin in the environment.^70^ Mercury poisoning causes the most concern among toxicologists because it primarily affects central nervous system (CNS) neurotoxicity^69,70^ and has been linked to neurodegenerative diseases such as neural stem cell dysfunction and neurodevelopmental abnormalities.^71^ This is particularly true during fetal development since the toxicity threshold is significantly lower, and neurotoxic effects are much more severe than they are in adults.^69^ Research conducted in Iraq indicated negative impacts on fetal development when the concentration of MeHg in maternal hair was about 20 μg/g.^72^ In addition, children who were exposed to maternal hair concentrations of MeHg ranging from 10 to 20 μg/g, have been reported to have memory problems.^73^ Another research study was conducted in New Zealand on a group of females who were exposed to 6 μg/g of MeHg during pregnancy and by examining their children at the age of four using the Denver test; their results were found to be abnormal compared to those of a control group with no history of MeHg exposure. Subsequently, using the Wechsler intelligence test at the age of six, lower performance for children who were exposed to MeHg was observed.^74^

The effects of prolonged exposure to organic mercury (MeHg) on individuals have been extensively investigated. MeHg poisoning can result in detrimental effects on the CNS, as evidenced by histological changes and clinical symptoms observed in affected individuals. These symptoms of poisoning, resulting from neurodegeneration and characterized by oxidative damage, encompass a range of impairments, including compromised motor coordination, dysfunction of the visual and tactile sensory systems, and, in severe cases, paralysis.^75,76^ Moreover, recent research has suggested that chronic, low-level exposure to mercury could potentially be a risk factor for motor function deficits, such as amyotrophic lateral sclerosis (ALS). A notable case report revealed an older man diagnosed with ALS, who had a history of chronic mercury exposure at his workplace. Further genetic testing unveiled a mutation in the TBK1 gene linked to ALS, providing a possible link between mercury exposure and neurodegenerative diseases.^77,78^ Additionally, a systems biology investigation conducted on hippocampus cells exposed to methylmercury has indicated substantial alterations in proteins associated with prolonged exposure. These findings shed light on potential connections between mercury exposure and neurodegenerative conditions such as Parkinson’s disease (PD) and Alzheimer’s disease (AD).^79^

In an animal study, mercury deposits were discovered in the lumbar region of the spinal cord of wild mice captured in the vicinity of a site of volcanic activity. These specimens exhibited the hallmark of neurodegenerative pathologies such as decreased axon calibre and axonal atrophy.^80^ The same research team reported in a separate study that mercury was accumulated in mice exposed to volcanic ash in the blood vessels and brain (i.e., white matter and hippocampus cells). This finding shows that long-term exposure to active volcanic environments leads to brain mercury accumulation, which can be a risk factor for human neurodegenerative diseases.^80^ In Amyloid β (Aβ)-induced memory impairments of AD, mercury significantly impaired spatial learning and memory. Mercury poisoning caused mitochondrial dysfunction that promoted spatial memory problems in rats. Mercury increased the generation of reactive oxygen species (ROS), MMP breakdown, mitochondrial enlargement, glutathione oxidation, lipid peroxidation, and damage to outer membranes, which furthered the harmful effects of Aβ on spatial memory and hippocampal mitochondrial function. Additionally, the rats’ hippocampi displayed increased ADP/ATP ratio and decreased cytochrome c oxidase (complex IV) activity.^81^ The worst-case scenario is that a study finds a connection between prior mercury exposure from eating seafood and a variety of later-life nervous system disorders, such as extrapyramidal impairment, sensory impairment, cranial nerve disturbances, gross motor impairment, neurocognitive deficits, and affective mood disorders.^82^

Largely through *in vitro* and *in vivo* research, the molecular processes underpinning MeHg-induced neurotoxicity have been elucidated. Cellular and molecular alterations in brain cells exposed to MeHg include the production of cytokines, oxidative stress, mitochondrial malfunction, Ca^2+^ and glutamate dyshomeostasis, and cell death pathways.^75^ The CNS is permanently damaged by methylmercury, a frequent and strong environmental neurotoxic that rapidly passes the blood-brain barrier. In an *in vitro* study, it was discovered that 3 μM of methylmercury caused cytotoxicity in neurons by ER stress, followed by the induction of programmed cell death (apoptosis) and cell death.^83^

The molecular mechanism of toxicity caused by inorganic mercury compounds includes disruption of cell membranes and affecting cell integrity and permeability. Subsequently, the inorganic mercury compounds start binding to thiol groups in proteins, leading to protein denaturation and dysfunction followed by oxidative stress by generating ROS, which is a similar mechanism to that of MeHg.^84^ The mercury compounds inhibit enzymes involved in antioxidant defense mechanisms and contribute to oxidative damage. Also, neuroinflammation by inorganic mercury triggers inflammatory responses in the central nervous system.^85^

Although research on the neurotoxicity of mercury compounds has made tremendous strides since the second half of the 20th century, Branco et al.^69^ asserted that there are still many open concerns about the toxicity processes. They summarized the results of extensive research that has been done in the last two decades on the molecular interactions of mercury that lead to neurotoxic effects, with a focus on the disruption of glutamate signaling and excitotoxicity brought on by mercury exposure as well as the interaction with redox-active residues like cysteines and selenocysteines, which is the basis for the disruption of redox homeostasis brought on by mercurial. The emergence of neurotoxicity in the CNS is influenced by the activation of microglia and astrocytes.^69^ Branco et al.^69^ proposed that future research should focus on the effects of low exposure levels, specifically the activation of immune cells in the CNS and neurodifferentiation as the basis of neurodevelopmental toxicity. This issue has been addressed in a study by Li et al.^86^ They discovered that mercury consumption had an impact on energy metabolism, the glutamate/GABA-glutamine shuttle, and neuroprotective cascades in astroglia at the molecular level. Neurodegeneration is facilitated or even caused by deficiencies in these astroglia pathways.

Branco et al.^69^ also emphasized that it is essential to consider how dietary factors (e.g., PUFAs and selenium) can reduce or eliminate these low-level mercury neurotoxicity effects. This is crucial for improving risk assessment methods and better understanding the causes of mercury neurotoxicity.^69^ Previously, Bjorklund et al.^70^ reviewed the molecular interaction between mercury and selenium in neurotoxicity. They hypothesized that selenium (Se) may be utilized as a neuroprotectant against mercury neurotoxicity as it creates stable coordination complexes with Hg. Its efficiency may be affected by the dosage of Se elements as well as their unique chemical structures, since their molecular interactions also entail interactions between Hg and different selenoproteins. In future, Bjorklund et al.^70^ suggested assessing the details protective effect more on adequate doses and suitable Se compounds, as inappropriate doses may cause the opposite effect. The type of Se compound used may also influence the result of inconsistency. In line with Branco et al.^69^ suggestion, Novo et al.^75^ agreed that efforts should be undertaken to limit the incidence of mercury poisoning and provide proper protection to individuals who are exposed. In such communities, monitoring and intervention measures, as well as the legal establishment of mercury reference doses and clinical intervention processes, are essential. Additionally, public knowledge of the effects of mercury poisoning is required to prevent unnecessary exposure.

**Figure 3:**
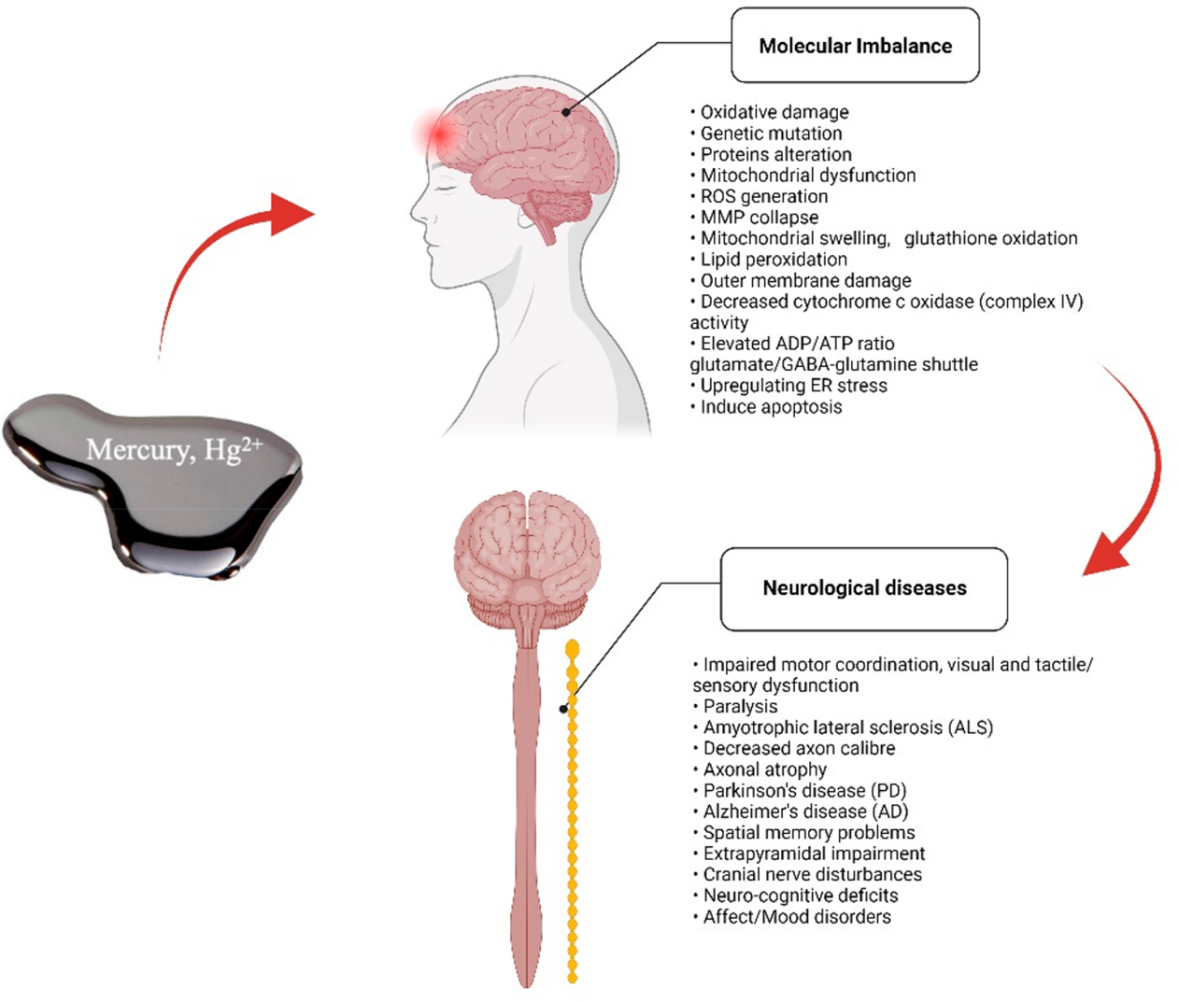
Toxic effects of mercury that contribute to neurological disorders via molecular imbalance.

The summative information about the neurological impairment is outlined in Figure 3 and tabulated in Table 2.

**Table 2:** Effects on Human Health and Associated Molecular Mechanisms

Disease	Molecular Mechanism	Related Impairment	References
Neurological diseases	Molecular imbalance:	• Neural stem dysfunction	Raposo et al.,^71^ Arrifano et al.,^76^ Bittencourt et al.,^77^ Magnavita et al.,^78^ Karri et al.,^79^ Navarro-Sempere et al.,^80^ Behzadfar et al.,^81^ Philibert et al.,^82^ Chung et al.,^83^ Li et al.^86^
	• Oxidative damage	• Neurodevelopmental abnormalities	
	• Genetic mutation	• Impaired motor coordination, visual and tactile/sensory dysfunction	
	• Proteins alteration		
	• Mitochondrial dysfunction		
	• ROS generation	• Paralysis	
	• MMP collapse	• Amyotrophic lateral sclerosis (ALS)	
	• Mitochondrial swelling, glutathione oxidation		
	• Lipid peroxidation	• Decreased axon calibre and axonal atrophy	
	• Outer membrane damage		
	• Decreased cytochrome c oxidase (complex IV) activity	• Parkinson’s disease (PD)	
	• Elevated ADP/ATP ratio	• Alzheimer’s disease (AD)	
	• Glutamate/GABA-glutamine shuttle	• Spatial memory problems and neurocognitive deficits	
	• Upregulating ER stress	• Extrapyramidal impairment	
	• Induce apoptosis	• Cranial nerve disturbances	
		• Mood disorder	
Genotoxicity and gene regulation	Molecular mechanism:	• Gene disturbance	Yang et al.,^58^ Crespo-López et al.,^87^ Ostrom et al.,^91^ Betti et al.,^96^ Crespo-López et al.,^97^ Lope et al.,^98^ Eke et al.^99^
	• Free radicals’ production	• Carcinogenesis	
	• Oxidative stress	• Glioma	
	• Microtubules		
	• Negatively affecting the process of DNA repair		
	• DNA damage		
	• Chromosomal aberrations		
	• Breakage of DNA strands		
	• Chromosomal disorders		
	• Disruption of chromosomal separation		
Immunogenicity	Molecular mechanism:	• Functional deficiency	Wada et al.,^101^ Rice et al.,^102^ Gardner et al.,^106^ Singh,^107,108^ Nyland et al.,^109^ Mishra,^176^ Liao et al.,^177^ Muhammad et al.^178^
	• Mercury inhibits PMN function by reducing its ability to eliminate foreign compounds by suppressing adrenocorticosteroids synthesis, which prevents proper stimulation of PMN formation	• Inflammatory problems	
		• Damaging tissues	
		• The generation of autoantibodies	
		• Deposition of immune complexes in vascular locations	
	• Trigger an immunological response in the central nervous system	• Weakening the immune system	
		• Hypersensitivity or allergy	
	• Modify immune cell formation and function	• Allergic disease	
	• Modulate interferon-gamma and interleukin-2 production	• Arthritis	
		• Autism	
	• Bacteria in the body release these toxic metals held inside them, leading to immune-related issues	• Attention disorder	
		• Eczema	
		• Epilepsy	
	• Escalated antinuclear autoantibodies as well	• Psoriasis	
	• Diminished anti-inflammatory cytokines	• Multiple sclerosis	
		• Schizophrenia	
		• Scleroderma	
Pregnancy and reproductive system damage	• Transfusion and defects mechanism:	• Ovulation disorders, tubal disease	Grandjean et al.,^74^ Henriques et al.,^116^ Hsi et al.,^117^ Bjo̷rklund et al.,^118^ Xue et al.,^119^ Gerhard et al.,^120^ Lei et al.,^121^ Pollack et al.,^122^ Choy et al.,^123^ Genuis et al.,^124^ Yoshida,^125^ Castoldi et al.,^127^ Mottet et al.,^128^ Dorea,^129^ Doja et al.^130^
	• Consumption of drugs that contain mercury-based preservative material	• Uterine abnormalities	
		• Infertility	
	• Mercury from the mother’s tissues flows easily through the placenta into the growing fetus during the periods of pregnancy	• Abortion	
		• Birth defects	
		• Menstrual disorders	
	• The inorganic form of mercury was verified to be transferred to the nursing infant via breast milk	• Congenital disorders	
		• Polycystic ovary	
		• Dysfunction of the thyroid gland	
	• Influences the endocrine system, leading to hormonal abnormalities in both men and women	• Neural tube anomalies	
		• Craniofacial deformities	
		• Retarded growth	
	• Reduction in the level of both progesterone and estradiol	• Reduce cerebral development of infants	
	• Reduce ovarian and testicular function	• Movement disorders	
	• Reduction of the transport of essential elements in the placenta	• Autism in infants and young children	
	• Reduce the number of nerve cells in the cerebral cortex of fetus		
Cancer promotion	Epigenetics implication:	• Lung	Zulaikhah et al.,^34^ Virani et al.,^136^ Pelch et al.,^137^ Brocato et al.,^138^ Maccani et al.,^139^ Goodrich et al.,^140^ Zefferino et al.,^141^ Intarasunanont et al.^179^
	• Histone modification	• Kidney	
	• RNA regulation	• Digestive system	
	• Alternative RNA splicing	• Nervous system	
	• RNA stability		
	• DNA methylation		
	• DNA repair		
	• Transcription		
	• Copy number of gene		
	• Transposon activation		
	• Inhibition of Gap junctional intercellular communications		
	• Immunosuppressive effects		
Cardiotoxicity	Cardiac mechanism implication:	Coronary heart disease	Guallar et al.,^142^ Counter et al.,^144^ Genchi et al.^145^
	• Cardiotoxicity: damage in the cardiac muscle	Cerebrovascular accident	
	• Pumping problem	• Myocardial infarction	
		• Hypertension	
		• Carotid artery obstruction	
		• Cardiac arrhythmias	
		- General atherosclerosis	
Pulmonary diseases	Mechanism of implication:	• Chemical pneumonitis	Rice et al.,^102^ Bjorklund et al.,^151^ Zulaikhah et al.,^152^ Bridges et al.^154^
	• Vapor inhalation can be absorbed into the lungs	• Dyspnea	
		• Cough	
	• Penetration to the blood barrier of the placenta and brain to distribute to the whole body	• Difficulty in breath	
		• Chest pain	
Renal diseases	Molecular implication:	• Nephropathy	Taux et al.,^162^ Geier et al.^165^
	• Minimize mRNA	• Hydronephrosis	
	• Downregulate aquaporins	• Acute pyelonephritis	
	• Alterations in kidney tissues	• Glomerulonephritis	
	• Severe interruption to the renal function	• Renovascular hypertension	
	• Histone post-translation modification		
	• DNA methylation		

### 4.2 Genotoxicity and Gene Regulation

Different species of mercury, including MeHg and the inorganic form of mercury, were concluded to be responsible for causing genotoxicity in humans by free radicals production, exerting oxidative stress, disrupting microtubules, and negatively affecting the process of DNA repair.^87^ Genotoxicity of chromosomes and disruption of DNA were detected in people exposed to high levels of mercury.^88^ In addition, a positive correlation between the breakdown of DNA molecules and exposure to high levels of mercury was recently observed.^89^ Factors, including the type of mercury species (Hg^0^, Hg^2+^, or MeHg) and organ that is investigated for mercury (i.e., urine, blood, hair), usually control the genotoxicity of mercury.^90^ In addition, the dose of mercury, as well as the xenobiotic defense of encoding genes, is reckoned to be a key factor in determining the genetic toxicity of mercury on human health.^90^ Also, it must be mentioned that MeHg led to gene disturbance and carcinogenesis, yet the actual mechanism still needs further investigation.^58^ MeHg was recently related to the high rate of incidence of Glioma, which is a tumor commonly arising from glial cells of the brain, representing around 80% of malignant brain tumors in US adults.^91^ MeHg and inorganic mercury compounds induce the generation of ROS within cells. ROS, such as superoxide anions and hydroxyl radicals, can cause oxidative damage to DNA bases, leading to DNA strand breaks, base modifications, and DNA adduct formation. Mercury has been shown to interfere with DNA repair mechanisms, including base excision repair and nucleotide excision repair. Mercury-induced genotoxicity may trigger apoptosis as a cellular response to severe DNA damage. In addition, mercury exposure can lead to genomic instability, characterized by an increased frequency of mutations and chromosomal aberrations.^9^

Several assays, including sister chromatid exchanges (SCE), chromosomal aberrations (CA), cytochalasin B blocked micronucleus test (CBMN), and single-cell gel electrophoresis, are frequently used to determine the genotoxic effect of mercury compounds (SCGE or alkaline comet assay).^9^ In this regard, Bucio et al.^92^ used the comet assay to investigate the effect of HgCl_2_ on a human fetal liver cell line (WRL-68) to prove the DNA damage by mercury compounds. So, they concluded that raising the mercury concentration and exposure duration increased the nucleus and DNA breakage rate. Cells treated with 0.5 μM and 5 μM of HgCl_2_ demonstrated an increase of 60% and 166% in nucleus damage, respectively, when compared with untreated control cells. Treatment of cells with 0.5 μM HgCl_2_ for a prolonged period of 7 days resulted in an increase of 200% in nucleus damage. Additionally, the comet assay was employed in another study that was carried out by Ben-Ozer et al.^93^ to identify the toxic effect of HgCl_2_ on human DNA. U-937, a cell line derived from cancerous cells isolated from a patient’s pleural effusion who had histiocytic lymphoma, was used in this study to demonstrate that there is a direct correlation between the incidence of DNA damage and the level of HgCl_2_. The exposure of U-937 cells to 0–100 μM of HgCl_2_ for 24, 48, and 72 h also suggested that raising the mercury concentration and exposure duration increased the amount of DNA damage. Moreover, the same assay was utilized to scrutinize the perilous impact of HgCl_2_ on the lymphocytes and the salivary gland cells in humans by detecting the single-strand breaks that resulted in the migration of DNA along with the partial DNA repair that exacerbates the issue.^94^ It was reported that increasing the concentration of HgCl_2_ in tissue cells resulted in a significant increase in DNA migration when compared to the negative control, with significant reductions in cell viability below 75% being observed when the tissue cells were treated with 50 μM of HgCl_2_.^94^ Concerning the toxic effect of mercuric nitrate on human genes, Lee et al.^95^ revealed that mercuric nitrate could produce endoreduplication at a concentration of 30 μM.

The genotoxic effect of organic compounds of mercury such as MeHg and dimethyl mercury, which are even postulated to be more toxic than the inorganic contaminants of mercury, in humans was also proved via their induction of structural and numerical chromosomal aberrations in lymphocytes as it was previously elucidated by Betti et al.^96^ Additionally, it results in escalating the frequency of sister chromatid exchanges in lymphocytes from blood cultures, breakage of DNA strands, chromosomal disorders, and disruption of their separation as elaborated in different research studies, including those conducted by Crespo-López et al.,^97^ Lope et al.^98^ and Eke et al.^99^ Exposure to MeHg was found to have a clear correlation with cytogenetic damage in lymphocytes at levels of hair mercury which are less than 50 μg/g.^100^

### 4.3 Immunogenicity

Mercury has been recognized for a long time to affect immune system function, most likely through its negative effects on polymorphonuclear leukocytes (PMNs). Mercury inhibits PMN function by reducing its ability to eliminate foreign compounds by suppressing adrenocorticosteroids synthesis, which prevents proper stimulation of PMN formation.^101^ Mercury has the capacity to alter immune cell development and function as well as the synthesis of interferon-gamma and interleukin-2 in the central nervous system.^102^ Exposure to mercury can induce oxidative stress and trigger inflammatory responses in immune cells by producing ROS. Mercury interferes with antioxidant defense mechanisms, such as glutathione metabolism. Subsequently, depletion of antioxidants can impair the ability of immune cells to neutralize ROS and protect against oxidative stress. Mercury can directly affect the function of various immune cells, including T cells, B cells, and macrophages.^103^ Moreover, altered cytokine production, impaired phagocytosis, and reduced lymphocyte proliferation are among the functional changes observed. Mercury exposure has been linked to autoimmune reactions and molecular mimicry, where immune responses may target self-antigens due to similarities with mercury-containing structures, resulting in the development of autoimmune diseases. Mercury has the ability to alter immunological signaling pathways, such as the mitogen-activated protein kinase and nuclear factor-kappa B (NF-κB) pathways.^104^ Genes involved in immunological responses can have their expression altered by deregulation of these pathways.^105^

**Figure 4:**
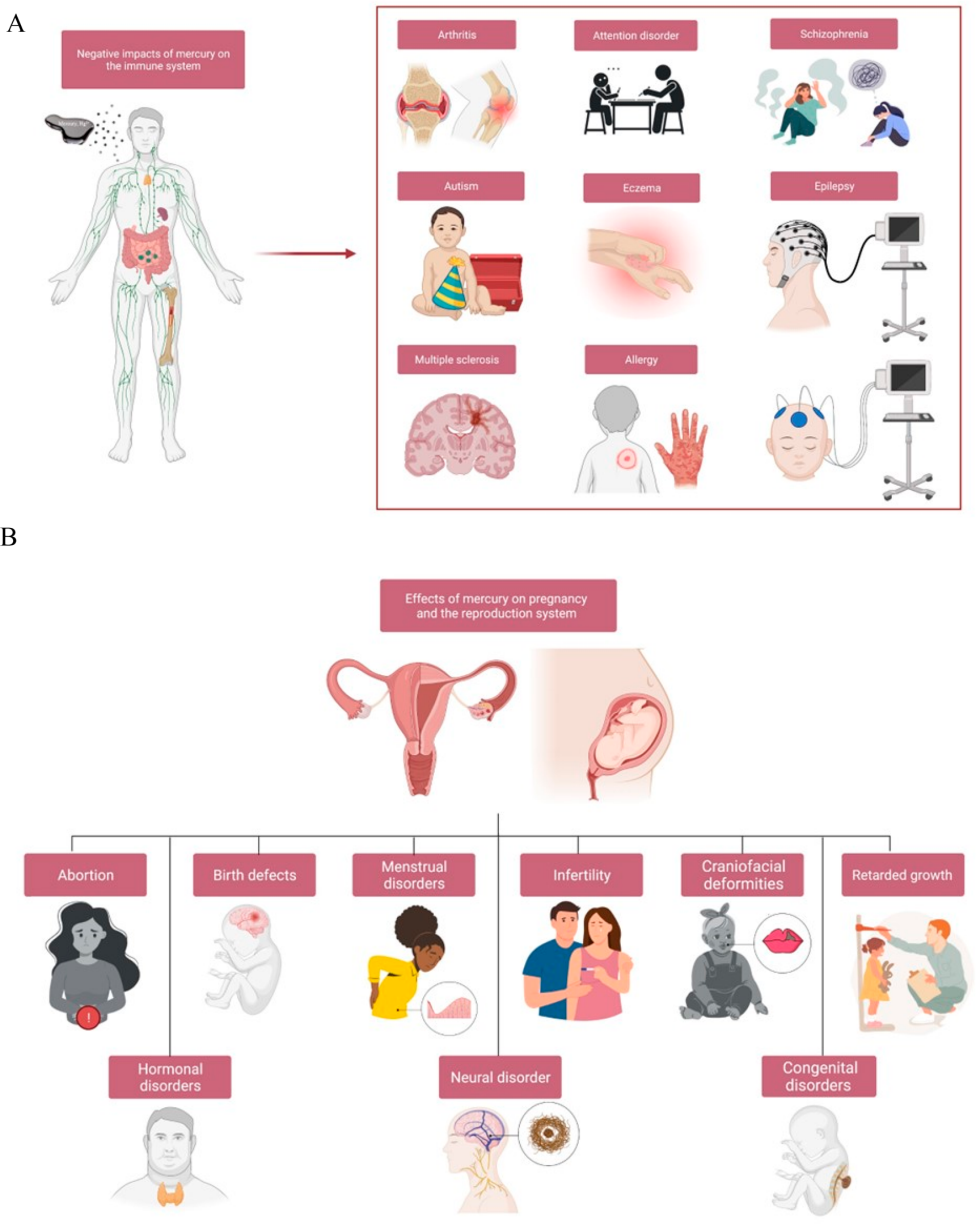
Negative impacts of mercury on the immune system (A) and effects of mercury on pregnancy and the reproduction system (B).

Consumption of mercury is frequently linked to higher amounts of bacteria, which are assumed to operate as a protective mechanism by absorbing excess mercury from the body. Antibiotics’ indiscriminate and quick eradication of these bacteria in people being continuously exposed to high levels of toxic metals, particularly mercury, could result in the release of these toxic metals held inside them and lead to immune-related issues such as allergic disease, arthritis, autism, attention disorder, eczema, epilepsy, psoriasis, multiple sclerosis, schizophrenia, and scleroderma^106−108^ as shown in Figure 4A.

There has been evidence linking increased mercury exposure levels to increasing levels of antinuclear autoantibodies and lower levels of anti-inflammatory cytokines, notwithstanding the paucity of studies on the harmful effects of mercury exposure on the human immune system.^109^ The form and dose of mercury, the route and timing of exposure, and the individual health circumstances affect how severe these immunological problems are. In a research study that was undertaken in Brazil,^110^ a range of hair mercury of 0.3–83.2 μg/g with an average dose of 8.6 μg/g was recorded for individuals who had worked with mercury in gold mining sites. Those individuals had a history of malarial infection that was 4.2-fold higher than that of individuals who had never worked with the metal. Also, the same study mentioned that exposure to mercury vapor, rather than MeHg, might be responsible for the malarial infection. However, no studies have been done to determine if exposure to mercury during critical periods of development, such as the prenatal period, alters the immune system’s response to mercury over the long term.^111^

### 4.4 Pregnancy and Reproductive System Damage

In recent years, the rate of infertility has substantially increased across the globe owing to different reasons such as the health issues of women’s age, ovulation disorders, tubal disease, and uterine abnormalities along with lifestyle factors including drinking alcohol and smoking as well as environmental stressors.^112−115^ However, the major influence was considered to be resulting from exposure to heavy metals, including mercury.^116^ The positive correlation between high concentrations of mercury in women’s blood and the incidence of infertility was previously concluded and confirmed. The same study reported that 80% of infertile women and 68% of pregnant women contained hair methylmercury concentrations exceeding the reference dose of 1 mg/kg established by the US Environmental Protection Agency, and this positively correlated with the daily methylmercury exposure dose.^117^ The prevalence of menstruation abnormalities among Hg-exposed women was linked to the number of years spent working in the dental field.^118^ In addition, other health issues were figured out to result from being exposed to mercury, including abortion, birth defects, menstrual disorders, and congenital disorders (Figure 4B).^119^ Hormonal disorders that usually lead to polycystic ovary and thyroid gland dysfunction are reckoned to result from high levels of mercury.^120,121^ Also, the reduction in the level of both progesterone and estradiol was related to the high mercury concentration.^122^

Regarding the negative impact of mercury on male fertility, it harmfully influences the endocrine system of males by affecting testosterone and subsequently exacerbating infertility rates.^123^ One reason for this adverse effect of mercury on human reproductive health is that it may be an endocrine disruptor, causing hormonal irregularities in both men and women that may impair fertility and cause a reduction in ovarian and testicular function.^116^

Mercury from the mother’s tissues flows easily through the placenta into the exceedingly susceptible growing fetus during the periods of pregnancy,^124^ leading to neural tube anomalies, craniofacial deformities, retarded growth, and other problems.^125^ Also, inflammation caused by mercury may contribute to reproductive dysfunction and complications during pregnancy.^126^ Additionally, it negatively affects the brain development of infants, consequently leading to cerebral and movement disorders in the following growth stages.^127^ The reduced number of nerve cells in the cerebral cortex, obvious diminution in the brain’s weight, and neural ailments were all accredited to mercury exposure.^74^ Also, it was proved that mercury constrains the transport of essential elements in the placenta.^128^ The inorganic form of mercury was verified to be transferred to the nursing infant via breast milk.^129^ It has to be mentioned that the incidence rates of autism in infants and young children significantly escalated in recent years owing to their exposure to high levels of the organic type of mercury (MeHg) and inorganic mercury through the mother’s blood and breast milk, respectively.^130^ Another reason that was concluded to be contributing to this incidence was the excessive consumption of drugs that contain thimerosol, which is a commonly used mercury-based preservative material in the production of pharmaceuticals.^131^ A research study was carried out in Hong Kong^123^ indicated the relation between the high blood mercury concentration, which was about 50 μg/L, and the infertility of both males and females was a result of consuming MeHg-contaminated food. Also, the same study revealed that males and females, even with MeHg concentrations of 40.6 and 33.2 μg/L, respectively, in their blood, were associated with fertility issues. In addition, vapors of inorganic mercury could affect the reproductive system of females who were exposed to a concentration of 0.01 μg/L by resulting in menstrual disorders and pregnancy problems.^132^

### 4.5 Cancer Progression

**Figure 5:**
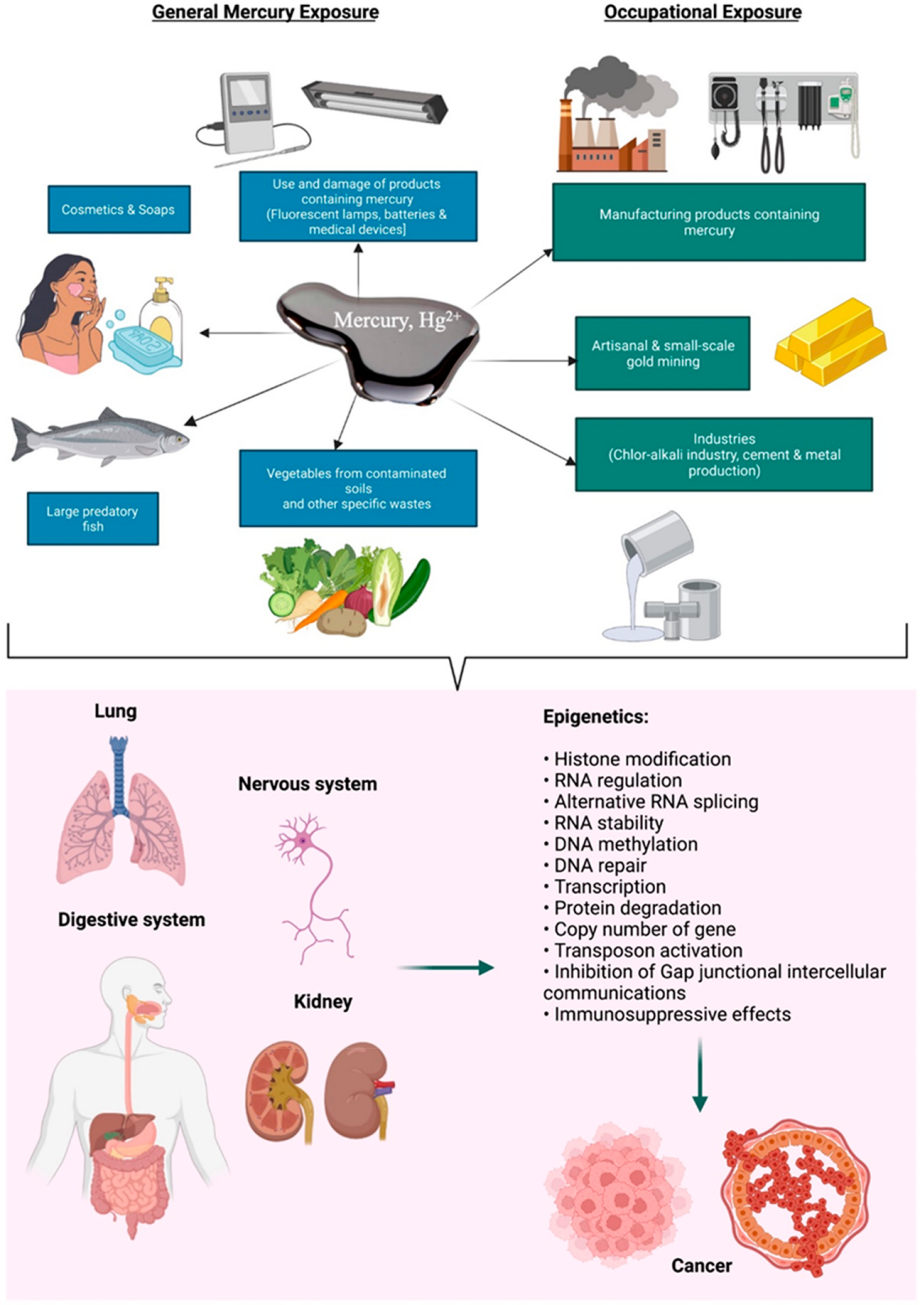
Sources of mercury and its toxicity toward cancer promotion via molecular imbalance (epigenetics).

Mercury is one of the potential agents to promote cancer development and progression, since it can be found particularly in the occupational environment (Figure 5). It has been shown that there is a significant correlation between occupational mercury exposure levels (as measured in toenail, hair and blood) and cancer risk and mortality.^133^ It might enter the human body differently, such as through inhalation, skin, and diet, ultimately affecting the lung, kidney, digestive, and nervous systems. The negative effects of mercury depend mostly on its speciation because different forms of mercury have different levels of bioaccessibility, bioavailability, and toxicokinetics. Even though elemental mercury is nontoxic (Hg^0^); however, the converted forms of mercury (methylmercury and Hg^2+^) are toxic and might accumulate in the body to cause a severe effect. These instances are potentiated to promote unexpected and nongenetical cancers. It has been reported that Hg^2+^ with thiol-compounds can form mercaptans and reduce the thiol-based antioxidants in cells, which is considered a potential step to induce cancer.^30^ A case study in Poland revealed the occurrence of leukemia among a group of farmers who used mercury-containing fungicides in their farmlands.^134^ The study indicated that the concentration of total mercury in the hair of those farmers was 1.24 mg/kg, compared to just 0.49 mg/kg in the hair of healthy individuals. Another case study in India showed that people with 3.67 μg/L of mercury in their blood developed renal cancer compared to healthy individuals with only 0.36 μg/L.^135^

As a well-known fact, epigenetics is a mandatory molecular complex event without any gene alterations. Mercury has been correlated to the interference of epigenetics by genetic modifications, including histone modification, RNA regulation, alternative RNA splicing, RNA stability, DNA methylation, DNA repair, transcription, gene copy number, and transposon activation. Hg species may cause oxidative DNA alterations and hinder DNA repair pathways due to their pro-oxidant activity.^133^ These processes in the gene alterations are the major causes of forming various cancers, mediated by mercury and other heavy metals/metalloids.^136−138^ Mercury has been found to induce global hypermethylation and hypomethylation in G-protein GTPase Rnd2.^139,140^ It has been hypothesized that mercury might be an epigenetic agent. In this context, Hg^2+^ helps to inhibit gap junctional intercellular communications and causes immunosuppressive effects. Mercury also mediates an upstream alteration of intracellular redox by specifically inhibiting antioxidant enzymes containing selenocysteines.^30^ Such an inhibition of cell communication can contribute to uncontrolled cell growth and proliferation, which are the main hallmarks of cancer development. Additionally, the immunosuppressive effects disturb the immune system’s ability to recognize and eliminate abnormal or cancerous cells.

Numerous epidemiological and experimental toxicological studies on the possible link between mercury exposure and cancer have been carried out in the past few decades. Even though high-dose mercury exposure has been linked to cytotoxicity, with primary damage to the nervous system as the most susceptible to Hg toxicity, low-dose mercury exposure may cause a proliferative response in normal and cancer cells through interference with the estrogen receptor, ERK1/2, JNK, NADPH-oxidase, and, potentially, Nrf2 signaling.^133^ An epidemiological investigation revealed a correlation between exposure to mercury with cancer causes.^141^ A connection between mercury exposure and acute leukemia was discovered in other investigations.^34^ On the other side, other animal experiments have shown that methylmercury exposure over time increased the growth of kidney tumors.^34^

### 4.6 Cardiotoxicity Mediated by Mercury Exposure

**Figure 6:**
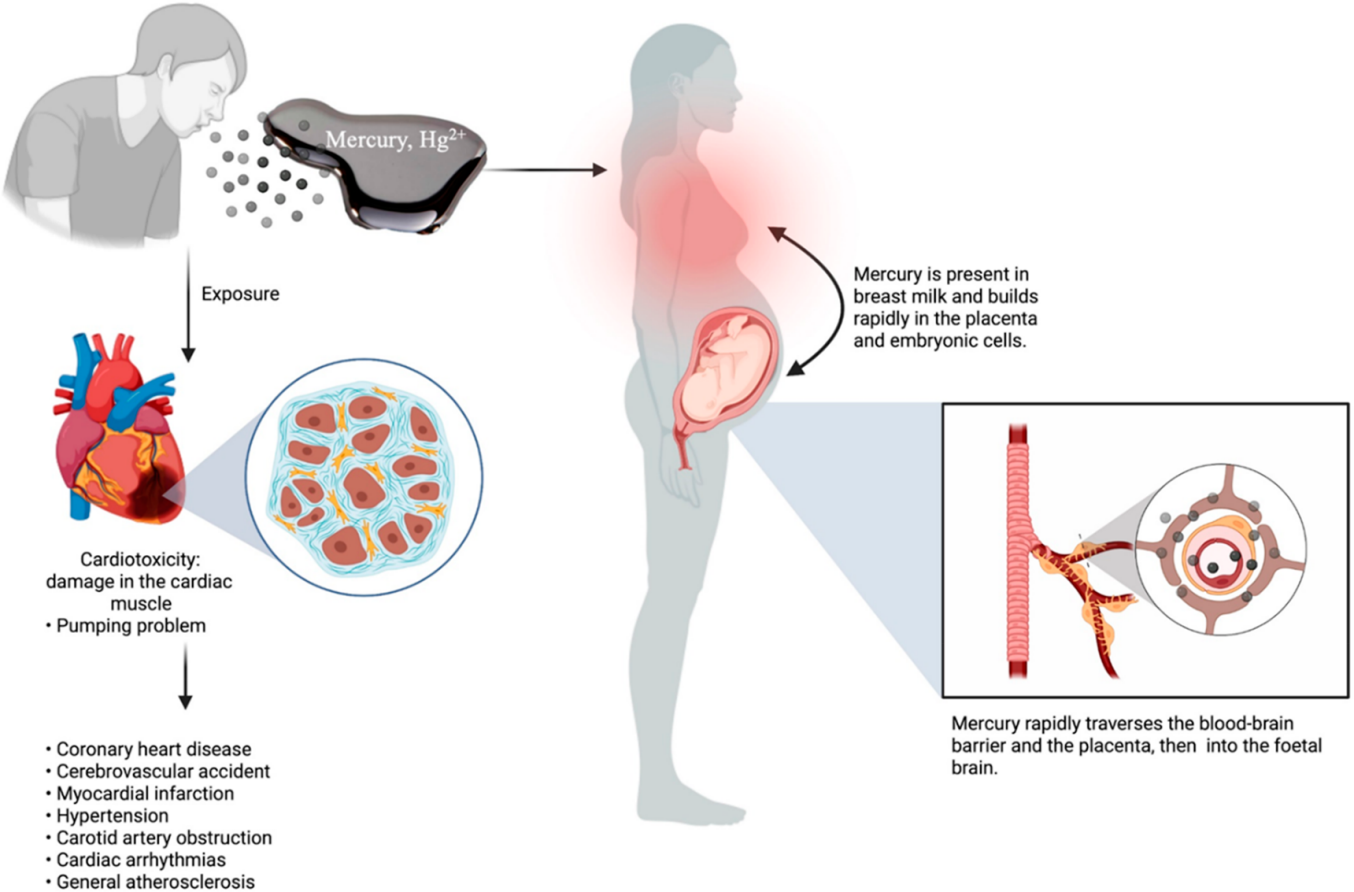
Cardiotoxicity mediated by mercury exposure.

Cardiotoxicity is primarily indicated by the damage to the muscle and ends up with a pumping problem in the heart. In the past decade, the impacts of mercury on the heart tissue have been noticed.^142^ World Health Organization (WHO) and Woods et al.^143^ stated that mercury-mediated toxicity is a fundamental issue associated with malfunctioning different human parts. Mercury toxicity is strongly associated with atherosclerosis in general, coronary heart disease, stroke, myocardial infarction, hypertension, carotid artery blockage, and cardiac arrhythmias (Figure 6).^144,145^ Also, mercury causes inflammation, which contributes to the progression of cardiovascular diseases by promoting endothelial dysfunction. Ion channels that are responsible for preserving cardiac electrical conductivity may be disrupted by mercury. Ion channel dysfunction can cause abnormal heartbeats.^146^ Mercury reduces the bioavailability of nitric oxide (NO), which inhibits vascular endothelial function. Subsequently, vascular deterioration is exacerbated by such an endothelial failure. Mercury disrupts mitochondrial function, leading to impaired energy production, cardiac dysfunction, and heart failure.^147^ The contractility of cardiac muscle cells is impacted by mercury’s interference with intracellular calcium homeostasis. Furthermore, heart inflammation is exacerbated by mercury because it activates pro-inflammatory signaling pathways, including NF-κB (nuclear factor kappa-light-chain-enhancer of activated B cells).^33^

Rice et al.^102^ revealed the systemic pathophysiology with toxicological effects of mercury on different human systems. The dose-dependent response of mercury exposure to cardiovascular health was noted by Roman et al.^148^ Among other heavy metals, mercury was noticed to be a direct-affecting compound to the cardiovascular and nervous systems. A correlation between the levels of mercury in urine and hair was found to be related to the levels of hematocrit and hemoglobin. Higher mercury levels in the hair and urinary display lower hematocrit and hemoglobin concentrations.^149^ Thus, proper public management of general mercury exposure should be given to prevent heart-related diseases and save a life. A recent study addressed the relation between cardiovascular diseases and exposure to mercury, in which the authors concluded that the risk of cardiovascular diseases occurrence begins to be high for the individuals who have a concentration of 2 μg/g of total mercury in their hair.^150^

### 4.7 Pulmonary Diseases

**Figure 7:**
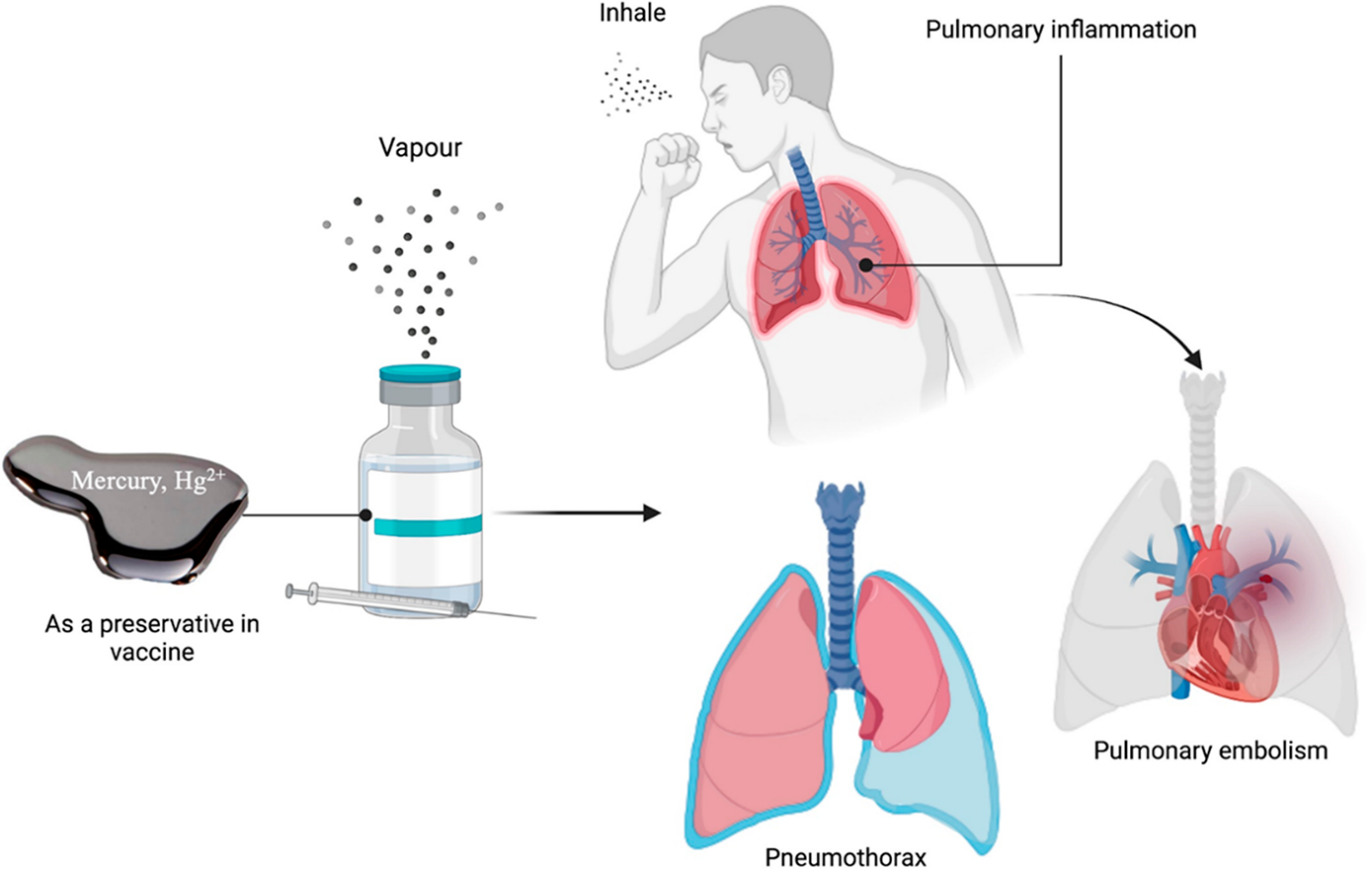
Cardiotoxicity mediated by mercury exposure.

There is a high possibility of scattering different metals into the pulmonary system along the pulmonary vasculature. The vapor formed from mercury inhalation at 80% can be absorbed into the lungs, followed by penetrating the blood barrier of the placenta and brain to distribute to the whole body.^151,152^ Mercury and other metals are also used to absorb from the gastrointestinal tract; on the other hand, the median mercury level in blood was noticed to be 0.73 ± 0.91 μg/L.^153^ Another source of mercury is an organic compound that contains mercury called “thimerosal” in use as a preservative with vaccine vials. Mercury-mediated pulmonary diseases are highly associated with the vaporized form of mercury (Hg^0^) that can be easily adsorbed from the lungs into the entire body. The liquid form of Hg^0^ has a higher chance of entering the gastrointestinal tract; however, it will not cause a toxic effect. Hg^0^ is used to oxidize and yield divalent mercury (Hg^2+^). CH_3_-Hg interacts with the thiolate molecules, such as cysteine, on the protein, facilitating the diffusion.^154^ The elemental forms of mercury vapor cause pulmonary-related issues such as chemical pneumonitis, dyspnea, cough, breathing difficulty, chest pain, asthma, and others (Figure 7). Mercury has the ability to influence both innate and adaptive immunity in the lungs via modulating immunological responses. Allergies and respiratory conditions may be exacerbated by immune system dysregulation.^155^ Mercury exposure can cause direct harm to the alveoli and epithelium lining of the lungs. Lung function may be negatively impacted by direct tissue damage, which can also hasten the fibrosis process. Fibrosis involves the excessive deposition of collagen in lung tissues, leading to impaired respiratory function.^156^ Rice et al.^102^ reviewed the toxicological effects mediated by mercury on different body organs associated with the lung, including Young’s syndrome, bronchitis, and pulmonary fibrosis. These incidents have permanently documented the negative consequences of mercury on human health. Overall, the prevention of vaporized mercury is needed to prevent the toxicological effect. A recent study in Indonesia^157^ indicated that adverse respiratory effects could be observed in individuals with a hair mercury concentration higher than 5 μg/g, while those with a concentration less than 1 μg/g are considered fine, and for those who have concentrations between 1 and 5 μg/g are in the warning levels.

### 4.8 Renal Diseases

**Figure 8:**
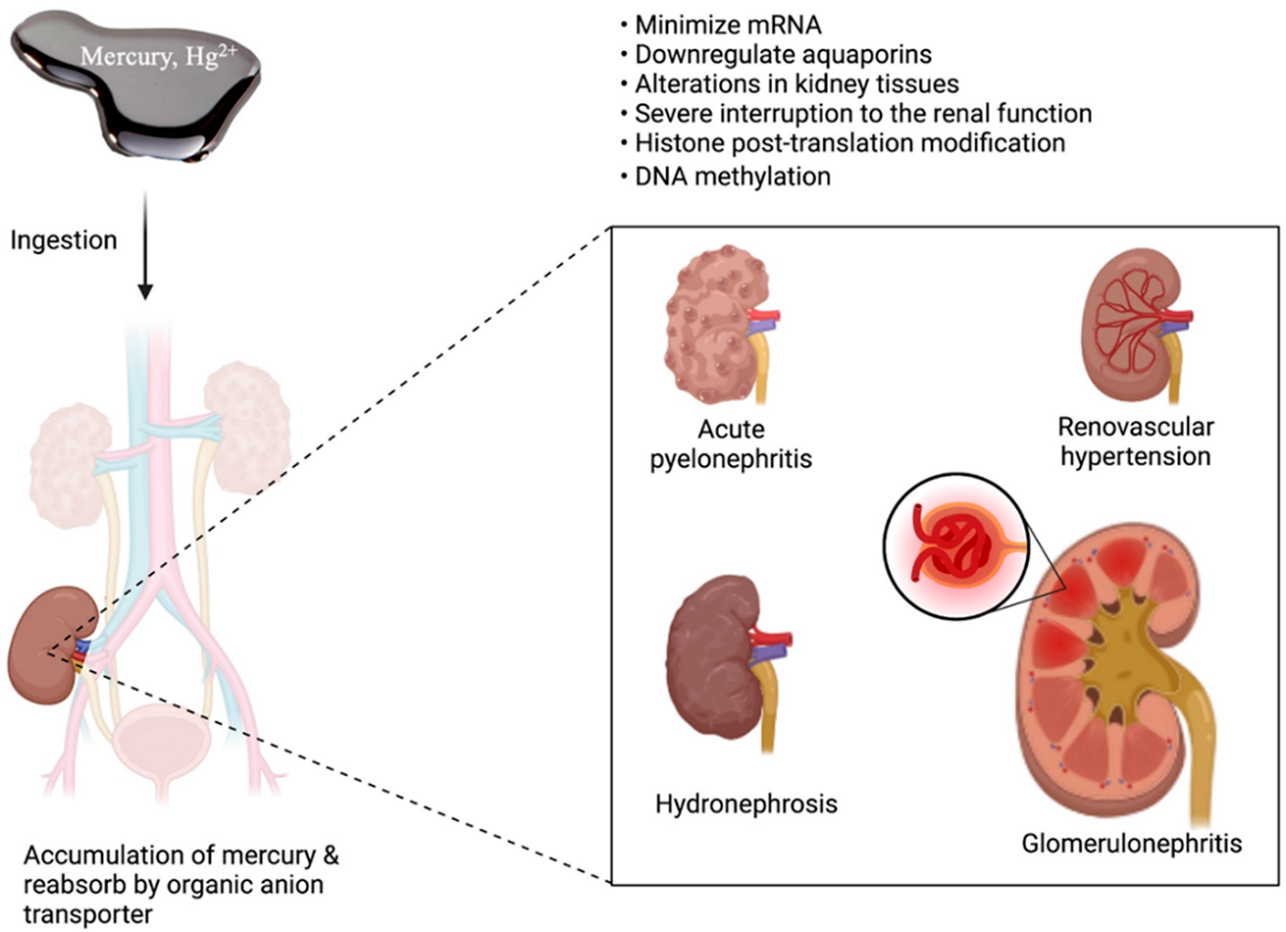
Renal diseases mediated by mercury exposure.

Kidneys are a primary organ targeted by inorganic mercury.^158^ It has been found that mercury-induced alterations are more severe in women compared to the effect on men.^159^ The WHO is worried about mercury as a health risk, especially the possible adverse effects on the kidneys, including autoimmune dysfunction and neurological symptoms.^160^ The primary issue with mercury is its half-life in the bloodstream as 2–4 days during excretion. In general, mercury absorption is relatively lower from the digestive tract; however, a more significant amount might enter the body by accidental or suicidal ingestions.^161^ Besides, elemental mercury exposure, such as that experienced by artisanal and small-scale gold mining workers, has been linked to kidney damage.^162^ In an earlier study, a relation between enhancing mercury exposure with elevated urinary mercury levels (18% to 52% among 8–18-year-old individuals),^163^ increased mercury body burden (5–10% increase in mercury-associated porphyrins),^164^ and other evidence was shown to increase kidney damage (Figure 8).^165^ It has been proposed that the threshold for adverse renal toxicity in adults at occupationally relevant air concentrations ranges from 25 to 30 μg/m^3^ of Hg vapors, which is equal to 35 μg/g of creatinine.^166^ Another study^167^ revealed that some renal disorders in a group of miners in Indonesia, such as proteinuria, which is a condition characterized by the presence of an abnormal amount of protein in the urine caused by high concentrations of mercury in urine (>7–273.3 μg/L).

In general, inorganic forms of mercury are accumulated in the kidneys that can be reabsorbed as Cys-S-Hg-SCys by organic anions transporters. In this study, further available data attested to the severity of renal disease with increased mercury presence.^168^ Akgül et al.^169^ investigated mercury adsorption in the animal body and noticed the alteration of the kidneys by histological examinations. The administration of mercury in the rat model impaired the expression of aquaporin in the gastrointestinal tract. It was observed that mercury exposure resulted in the downregulation of aquaporins 3 and 7 at the mRNA and protein levels, as well as aquaporin 8 at the mRNA level. Consequently, the reduced epithelial cell osmotic water permeability could impair the osmotic water equilibration and active water transport, resulting in fatal fluid accumulation, which could ultimately lead to kidney damage.^170^ On the other hand, it was registered that there was no association between hypertension and mercury in the blood but an inverse relation with urinary mercury.^171^

The epigenetic alterations in kidney tissues also severely interrupt renal function. Histone post-translation modification and DNA methylation are the highly noticed kidney epigenetic alterations by mercury exposure.^172^ Rice et al.^102^ reviewed the renal toxicological effects of mercury exposure on other organs. They found that there might be a release of mercury through urine and feces as the wastes, the remaining mercury amount still affecting the renal system. Thus, routine monitoring of renal function and urine analysis for heavy absorption and discard might help in prevention.

In addition, the minimal dosage of mercury that can cause different adverse effects varies depending on the specific effect and the exposure duration. Its toxicity in humans depends on various factors such as the chemical form of mercury, dosage, age of people exposed, length of exposure, entry into the body, fish diet, and consumption of seafood.^34^ Exposure to high concentrations of methylmercury or mercury vapor can cause damage to the brain, kidneys, and developing fetus. According to Li et al.,^173^ The United States Environmental Protection Agency (EPA), Food and Drug Administration (FDA) and the Occupational Safety and Health Administration (OSHA) have set a guideline for mercury exposure in drinking water (2 μg/L or 2 ppb), seafood consumption (1 μg/g or 1 ppm) and per cubic meter of workplace air (0.1 mg/m^3^ with 0.05 mg/m^3^ of mercury vapor for 8-h shifts and 40-h work weeks).

In summary, mercury exposure in its various forms poses significant hazards to human health. It mainly affects the CNS and has been linked to neurodegenerative diseases, particularly during fetal development. Prolonged exposure to organic mercury such as MeHg can result in impaired motor coordination, visual and tactile dysfunction, and paralysis. Mercury compounds induce genotoxicity, disrupting DNA repair processes and leading to chromosomal aberrations. Immunotoxic effects include immune-related diseases and hypersensitivity. Pregnant women exposed to mercury can experience infertility, birth defects, and negative effects on the fetus’s brain development. Additionally, mercury exposure has been linked to cancer progression, cardiotoxicity, pulmonary diseases, and renal disorders. Epigenetic alterations and histone modifications play significant roles in mercury-induced toxicological effects in various organs. Minimizing exposure to mercury is of utmost importance and can be achieved by the avoidance of contaminated food sources, implementation of appropriate protective measures in work settings, and adherence to rules outlined by health authorities. Therefore, preventative measures and risk assessments are essential to mitigate mercury exposure’s adverse effects on human health.

Quantitative data regarding the minimum dosages at which various adverse effects become evident in cases of mercury exposure are of paramount importance in understanding the extent of toxicity. Several studies have contributed valuable insights by identifying these critical thresholds. For instance, research has shown that even relatively low levels of MeHg exposure, such as 10 parts per billion (ppb) in maternal hair during pregnancy, can lead to adverse neurodevelopmental outcomes in children.^174^ Similarly, the WHO has established a provisional tolerable weekly intake (PTWI) for methylmercury at 1.6 μg/kg of body weight, emphasizing the significance of setting exposure limits to prevent toxicity.^175^ By summarizing such quantitative data, this review aims to provide a clearer perspective on the toxicological aspects of mercury and its associated health implications.

## 5 Ecotoxicity

Trophic cascades are ecological phenomena that happen when one trophic level of a food chain changes, having an impact on subsequent trophic levels in the process.^180^ Trophic cascades can happen across a minimum of three feeding levels, and they typically do so over three levels, while there is evidence of four and five-level cascades as well.^180,181^ Top-down and bottom-up are two different forms of trophic cascades. When predators are successful enough in their predation to decrease the abundance or change the behavior of their prey, the next lower trophic level is released from predation, which is known as a top-down cascade. When the abundance or productivity of a primary producer changes and has an impact on the abundance or productivity of the subsequent higher trophic level, this is known as a “bottom-up cascade”.

The bioaccumulation of mercury in higher trophic levels in terms of the concentration buildup in the tissues of organisms representing these higher levels is one important factor that might start top-down trophic cascades.^182^ Consequently, top predators lose some of their capacity to regulate the populations of their prey when mercury exposure has detrimental effects such as neurological issues and reproductive impairments. This may potentially change the structure and composition of entire ecosystems, such as population expansions of herbivores that overgraze or overuse primary producers. As a result, the abundance and distribution of species within an ecosystem, as well as their interactions, could be affected, leading to the disruption of ecological balance and the loss of biodiversity,^183^ which constitutes the most dangerous threat to humanity, even more than climate change.

It has to be mentioned that investigating the trophic cascades is a complex phenomenon that includes many different factors and processes, which must be fully covered to produce reliable data. One such case is a recent study undertaken by Seco et al.^184^ to study the biomagnification of mercury in the Scotia seafood web of the Southern Ocean over a period of nine years. The authors unexpectedly found that the concentration of mercury in a top predator (seabirds) increased over the nine years, whereas the mid-trophic levels (squid and myctophid fish) that feed on krill, which is a low mercury-containing species, showed a lower concentration after the same period. So, the authors concluded that in a condition where there is a scarcity of krill representing a bottom-up cascade, there would be a shift to different prey with higher Hg concentration, resulting in the high Hg concentrations detected in seabirds.

The toxicity of Hg simultaneously harms all living creatures and our sphere.^185^ The soil microbial community heavily influences the bioavailability of nutrients required for plant growth. Generally, among different biota, microorganisms are more sensitive to heavy metal stress.^186^ Regarding the literature, Hg has been shown to impede soil microbial activities such as the nitrification process and soil (enzymes/respiration) activities.^42,187^ The nitrification and urease processes in various soils have been demonstrated to be sensitive to Hg in comparable ways. EC_*x*_ is a semiquantitative approach that determines the level of Hg and has major deleterious impacts on microbial functioning.^188^ Mahbub^189^ reported a decrease in 20.0% of bacterial diversity, ammonia oxidizers, and nitrifiers with EC_20_ values of 4.4 and 11.1 mg Hg/kg soil, in neutral soil (i.e., pH = 7.6, organic carbon = 2.0%) and alkaline soil (i.e., pH = 8.5, organic carbon = 2.2%), respectively. The maximum permitted value of mercury in the soil is 0.5–5.0 mg /kg.^190^ Excessive Hg levels in soil cause acute toxicity in plants and jeopardize the sustainability of the ecosystem. Hg^2+^ ions in both inorganic and organic mercury compounds are primarily responsible for plant toxicity. Hg^0^ has a limited affinity toward cellular ligands and can only be harmful upon the oxidation to Hg^2+^ inside the cell. The Hg-induced plant toxicity is defined as suppressing plant growth as well as the yield of biomass production,^191^ negatively impact the efficacy of the photosynthetic process,^192^ deficiency of nutrients,^193^ oxidative stress,^194^ genotoxicity,^195^ and peroxidation of lipids.^196^ Hg^2+^ ions have a great affinity toward sulfur-containing groups, disrupting practically every activity involving essential or nonprotected proteins. Hg has been frequently listed to reduce the plant tissue content of chlorophyll, water, and minerals. Hydroponic cultures fed with different Hg dosages ranging from 5.0 to 80.0 g mL^–1^ were used to study the effects of Hg on Jatropha curcas plants.^197^ The findings revealed a decrease in biomass, reduced development, and suppression in photosynthesis. The exposure of plants to greater doses of Hg disrupted the chlorophyll concentration and net photosynthetic rate.^198^

Additionally, research was done on how mercury affected the development of the *Solanum lycopersicum* plant.^199^ Plants improved in germination rate, root length, early blooming, plant height, pollen viability, and chlorophyll content at low Hg concentrations. Contrarily, higher concentrations of Hg slowed and restrained the plant growth. Moreover, the nutrient imbalance is regarded as a toxicity marker for Hg exposure.^200^ The lipid membrane constituents of *Medicago sativa* were harmed in hydroponics conditions.^201^ Hg has also been associated with chlorotic and necrotic signs, as well as stunted growth.^202^ Greater Hg concentrations caused ultrastructural abnormalities in *Vigna radiata L,* such as nodule deformation, cell collapse, and reduction in the intercellular gaps.^203^

Invertebrates such as marine arthropods, worms, and *Drosophila* exposed to Hg have suffered undesirable severe impacts on locomotion, growth, eating, poor prey acquisition, and promotion of development defects in embryos. The worm (*C. elegans*) was dramatically affected by MeHg and HgCl_2_ exposure, which reduced movement, growth, and feeding and caused mortality.^204^ When *Drosophila* embryos were exposed to MeHg, they developed more slowly and had problems with patterning, positioning, and maturation of neurons and glia.^205^ Overall, the findings suggest that invertebrates, mostly those in their early life stages (eggs, embryos, and larvae), are more vulnerable to Hg exposure, but the exact mechanism by which Hg causes developmental abnormalities in embryos is unknown. The negative consequences of MeHg on several vertebrate species (i.e., amphibians, birds, fish, reptiles, and mammals) have been reported.^206−208^ Although there are significant variations in sensitivity among species toward Hg, literature displayed that the vertebrates’ exposure to MeHg and HgCl_2_ is associated with endocrine disruption,^209^ physiological malfunctions of liver/kidney,^210^ embryotoxicity,^207^ neurotoxicity,^211^ and changes in the reproductive habits.^212^

Overall, Hg’s toxic effects span across various organisms, highlighting the need for understanding and mitigating mercury contamination as it is negatively affecting soil microbial communities, hindering essential processes like nitrification and enzymatic activities. Additionally, it is responsible for plant toxicity, leading to suppressed growth, reduced biomass production, impaired photosynthesis, and nutrient deficiency. Vertebrate species are also affected, exhibiting endocrine disruption, liver/kidney malfunctions, embryotoxicity, neurotoxicity, and changes in reproductive habits.

## 6 Role of Education, Training Programs, and Social Media in the Phase-out of Mercury from Different Industries

The role of education in the phase-out of mercury is multifaceted and crucial for achieving sustainable and environmentally friendly practices. The key aspects of how education contributes to this goal include awareness and knowledge building, which serves as a primary tool for raising awareness about the environmental and health impacts of mercury.^213^ Also, it helps stakeholders such as industry professionals, policy makers, and the public understand the sources, pathways, and risks associated with mercury exposure.^214^ Capacity building is another aspect that provides skills and knowledge about mercury-free alternatives and safer practices that lead to a smoother transition away from mercury-dependent processes. Education facilitates the transfer of knowledge regarding mercury-free technologies and processes. It ensures that advancements and innovations in alternative methods are communicated and adopted by industries. Education facilitates the transfer of knowledge regarding mercury-free technologies and processes, and it ensures that advancements and innovations in alternative methods are communicated and adopted by industries.^215^ In addition, education provides the foundation for understanding and supporting government policies aimed at reducing mercury use. It enables stakeholders to engage in informed discussions and advocate for policies that align with sustainable practices. Education results in communities becoming key stakeholders in the movement to phase out mercury, ensuring that their voices are heard in discussions and actions related to environmental protection. Another factor is collaborative efforts, which can enhance the exchange of best practices, research findings, and successful case studies, accelerating the global transition to mercury-free technologies.

Training programs focus on developing the skills of professionals, workers, and technicians involved in industries using mercury. This includes training in the use of alternative technologies, safe handling practices, and understanding the environmental and health implications of mercury exposure.^216^ Informed individuals are more likely to support and actively participate in phase-out initiatives. Training sessions create a platform for knowledge exchange and discussions of best practices. In addition, training programs are often designed as ongoing initiatives to support continuous improvement via regular updates on technological advancements, safety protocols, and environmental best practices.

One of the case studies in which training programs were initiated by the United Nations to tackle the environmental issue of using mercury is a 3-year campaign from 2005 to 2008 in Tanzania, which is one of the fastest-growing mining countries in the last few decades.^216^ The initiative focused on enhancing the capabilities of community laboratories by providing training and necessary resources. The goal of this project was to facilitate ongoing monitoring even after the project’s initial phase, fostering understanding and awareness of the risks related to amalgamation among miners, government entities, and the broader public. The project aligned with the principle of advocating for cleaner and more efficient technologies, aiming to reduce adverse environmental effects while simultaneously enhancing income, health, and safety.^217^ The execution of the program took place in different stages, with the coordinators working in conjunction with community leaders to promote a comprehensive training approach aimed at fostering a shift in behavior among miners. In the case of Tanzania, raising awareness was not enough. In fact, providing practical demonstrations of technologies, exposure to alternative methods, and the establishment of trust to modify their practices was required. To address this, the United Nations team advocated for locally managed “mobile training units” to actively engage miners. Additionally, a manual for training artisanal and small-scale gold miners was created to offer guidance on cost-effective solutions. Brochures were crafted in Swahili, and local cartoonists contributed visuals, creating discussion materials that addressed various challenges, including mercury exposure, HIV/AIDS prevention, and other community-specific concerns. Not only did the program target gold miners, but it also included other community groups such as mercury dealers, family members of miners, district politicians, officers from the Ministry of Mines, official health workers, bank representatives, mining company representatives, and others. One of the major outcomes of this program was the legalization of gold miners, to stop the illegal and uncensored operations, along with providing financial support to help the poor workers in this field and also to accelerate the phase-out of mercury in these operations.

Information shared on social media reaches a broad audience, including industry professionals, policy makers, researchers, and the general public. Educational content, such as infographics, articles, and videos, which all have become available on different online platforms such as Google and YouTube, helps disseminate knowledge and promotes a better understanding of the issues related to mercury use. Establishing online communities and forums enables open discussions, knowledge exchange, and the sharing of experiences. It creates a platform for collaboration and encourages community-driven initiatives to address mercury-related challenges.^213^ Campaigns and movements initiated on social media platforms such as Facebook and Twitter can gain momentum quickly, drawing attention to the need for mercury phase-out. Activists and advocates can mobilize support, influencing public opinion and urging policy makers to take action.^218^ Moreover, social media serves as a channel for sharing success stories, best practices, and case studies related to the phase-out of mercury. It provides a space for showcasing successful transitions to mercury-free technologies. Over the past few years, social media has started to take the lead in the dissemination of updates, alerts, and news related to various issues, which could be extended to cover the news related to mercury regulations, technological advancements, and events.

To sum up, the bottom-up approach to the phase-out of mercury in various industries using education, training programs, and social media platforms is often considered more effective than the top-down approach due to its emphasis on grassroots involvement and community engagement rather than modifying the laws, policies, and regulations that are imposed from higher administrative levels down to the local or industry-related communities. In the bottom-up approach, the initiative begins at the local level, involving communities, workers, and stakeholders who are directly impacted by mercury use.^219^ This method recognizes the importance of local knowledge, practices, and concerns, allowing for tailored and context-specific solutions. By actively involving miners, industry workers, and community members in decision-making processes, the bottom-up approach fosters a sense of ownership and commitment. This community-driven strategy is more likely to be sustainable, as it addresses the unique challenges faced by different regions and industries. Additionally, the bottom-up approach promotes education, awareness, and behavioral changes within the affected communities, contributing to long-term success in reducing mercury use. Ultimately, the bottom-up approach recognizes the significance of local perspectives and actively involves those directly affected, making it a more inclusive and impactful strategy for the phase-out of mercury. All in all, social, environmental, institutional, and economic pillars should be simultaneously considered and integrated to achieve the successful phase-out of mercury.

## 7 Conclusion

As one of the most toxic elements on the earth’s surface, mercury’s toxicity was chosen to be investigated in the present study. Mercury typically exists in various forms, but MeHg has been deemed the most toxic. Approximately 90% of mercury compounds are emitted by human activities, such as mining operations, which account for the vast majority of mercury emissions. In an effort to lessen mercury’s harmful effects on both human health and the environment, the Minamata Convention on mercury was signed in October 2013 and came into force in 2017. Adsorption was shown to be the most efficient way to remove mercury from various ecosystems, despite employing a number of other techniques. Additionally, it has been proven that carbon-based compounds, such as carbon nanotubes and biochar, are very successful in the techniques used to detoxify mercury.

In addition, the primary properties of mercury element and its compounds have been investigated, including inorganic forms of mercury (elemental mercury Hg^0^, mercurous mercury Hg_2_^2+^, and mercuric mercury Hg^2+^) as well as organic mercury and its most prevalent forms, including methyl mercury, dimethyl mercury, and phenyl mercury. Additionally, the physicochemical properties of the most prevalent Hg compounds were detailed.

Even though there are few studies examining the dangers of mercury exposure to the ecosystem, genotoxicity, gene regulation, and the human immune system, Hg harms all living things and our environment. It has been discovered that higher mercury exposure is associated with elevated antinuclear autoantibodies and decreased anti-inflammatory cytokines. In addition, it was found that Hg causes human genotoxicity by producing free radicals, causing oxidative stress, disrupting microtubules, and negatively affecting the DNA repair process. The hazardous effect of mercury organic compounds such as methyl and dimethyl mercury on human genes has been established. In addition, there is a correlation between the increased rates of male and female infertility and exposure to high mercury concentrations.
